# Singlet oxygen induces cell wall thickening and stomatal density reducing by transcriptome reprogramming

**DOI:** 10.1016/j.jbc.2023.105481

**Published:** 2023-11-20

**Authors:** Zhong-Wei Zhang, Yu-Fan Fu, Xin-Yue Yang, Ming Yuan, Xiao-Jian Zheng, Xiao-Feng Luo, Meng-Yao Zhang, Lin-Bei Xie, Kai Shu, Steffen Reinbothe, Christiane Reinbothe, Fan Wu, Ling-Yang Feng, Jun-Bo Du, Chang-Quan Wang, Xue-Song Gao, Yang-Er Chen, Yan-Yan Zhang, Yang Li, Qi Tao, Ting Lan, Xiao-Yan Tang, Jian Zeng, Guang-Deng Chen, Shu Yuan

**Affiliations:** 1 College of Resources, Sichuan Agricultural University, Chengdu, China; 2 Key Laboratory of Investigation and Monitoring, Protection and Utilization for Cultivated Land Resources, Ministry of Natural Resources, Chengdu, China; 3 College of Life Science, Sichuan Agricultural University, Ya'an, China; 4 School of Ecology and Environment, Northwestern Polytechnical University, Xi'an, China; 5 Laboratoire de Génétique Moléculaire des Plantes and Biologie Environnementale et Systémique (BEeSy), Université Grenoble Alpes, Grenoble, France; 6 Sichuan Provincial Academy of Natural Resource Sciences, Chengdu, China; 7 College of Agronomy, Sichuan Agricultural University, Chengdu, China

**Keywords:** ABI4, cell wall, singlet oxygen, stomatal development, transcriptome reprogramming

## Abstract

Singlet oxygen (^1^O_2_) has a very short half-life of 10^−5^ s; however, it is a strong oxidant that causes growth arrest and necrotic lesions on plants. Its signaling pathway remains largely unknown. The *Arabidopsis flu* (fluorescent) mutant accumulates a high level of ^1^O_2_ and shows drastic changes in nuclear gene expression. Only two plastid proteins, EX1 (executer 1) and EX2 (executer 2), have been identified in the singlet oxygen signaling. Here, we found that the transcription factor abscisic acid insensitive 4 (ABI4) binds the promoters of genes responsive to ^1^O_2_-signals. Inactivation of the ABI4 protein in the *flu*/*abi4* double mutant was sufficient to compromise the changes of almost all ^1^O_2_-responsive-genes and rescued the lethal phenotype of *flu* grown under light/dark cycles, similar to the *flu*/*ex1*/*ex2* triple mutant. In addition to cell death, we reported for the first time that ^1^O_2_ also induces cell wall thickening and stomatal development defect. Contrastingly, no apparent growth arrest was observed for the *flu* mutant under normal light/dim light cycles, but the cell wall thickening (doubled) and stomatal density reduction (by two-thirds) still occurred. These results offer a new idea for breeding stress tolerant plants.

In plants, reactive oxygen species (ROS) are produced continuously as byproducts of multiple metabolic pathways and are localized in multiple cellular compartments. Under normal conditions, ROS are scavenged by various antioxidative defense systems (1, 2). The equilibrium between the generation and the scavenging of ROS may be perturbed by many environmental stresses. To date, research on the physiological activities of ROS in plant cells has mainly been restricted to hydrogen peroxide and superoxide, which are released upon abiotic and biotic stress and work as signaling molecules to regulate various processes, such as stomatal behavior, programmed cell death, and pathogen defense (1, 2).

In photosynthetic organisms, excited chlorophylls or tetrapyrrole intermediates can stimulate the formation of singlet oxygen (^1^O_2_) upon illumination; a highly toxic molecule that in addition to its damaging nature acts as a crucial signaling molecule. ^1^O_2_ signaling has been shown to interact with the signal cascades of other ROS, lipid hydroperoxide-derived reactive electrophile species, and oxidized carotenoids, which may induce programmed cell death (3). ^1^O_2_ is responsible for more than 80% of nonenzymatic lipid peroxidation (4). The FLU (fluorescent) protein is a nuclear-encoded plastid protein that plays a key role in the negative-feedback control of chlorophyll biosynthesis (5). Inactivation of this protein in the *flu* mutant leads to the over-accumulation of free protochlorophyllide (Pchlide), which may work as a potent photosensitizer. Thus, the *flu* mutant generates ^1^O_2_ in plastids in a controlled but noninvasive manner. Immediately after the release of ^1^O_2_, mature *flu* plants stop growing, whereas young seedlings bleach and die, when grown under dark/light cycles (5). Inactivation of the plastid proteins executer1 (EX1) and executer2 (EX2) attenuate the extent of ^1^O_2_-induced upregulation of nuclear gene expression, thereby reversing the growth arrest and seedling lethality of the *flu* mutant under light/dark cycles (6, 7).

Under severe light stress, the signaling is initiated independently of EX1 by singlet oxygen that is thought to be generated at the acceptor side of active photosystem II (PSII) within the core of grana stacks. The second source of ^1^O_2_ formation was found in the grana margins, close to the place of chlorophyll biosynthesis, where EX1 is localized and the disassembly of damaged PSII and reassembly of active PSII take place (8). The initiation of ^1^O_2_ signaling in grana margins depends on EX1 and the ATP-dependent zinc metallo-protease FtsH2 (9). Alternatively, ^1^O_2_ could oxidize β-carotene to release β-cyclocitral, which has emerged as a ^1^O_2_-induced stress signal in plants (10).

Despite these research progresses, little is known about how and where the ^1^O_2_ signals are sensed and transmitted to the nucleus, and no nuclear signaling factor has been identified. The ^1^O_2_ signal is a type of plastid retrograde signal (2, 3, 6, 7), in which plastid genomes uncoupled 1 (GUN1) protein and nuclear Apetala 2 (AP2)–type transcription factor abscisic acid insensitive 4 (ABI4) may be involved (11). A previous study demonstrated that the *gun1* mutation did not prevent the ^1^O_2_-mediated bleaching and cell death response of the *flu* mutant grown under light/dark cycles (12). Thus, the possible role of ABI4 in ^1^O_2_ signaling was investigated in this study. Previous studies suggested that ABI4 may function as a master switch, required for the regulation of nuclear genes in response to developmental cues (such as carbon source changes), environmental stress tolerance (responsive to abscisic acid, ethylene, and jasmonic acid), as well as chloroplast retrograde signaling (*e.g.*, norflurazon or lincomycin-trigged signals) (11, 13). In this report, we found that the inactivation of ABI4 was sufficient to abrogate ^1^O_2_-mediated signaling, resulting in *flu* mutant survival. In addition to cell death, we reported for the first time that ^1^O_2_ also induces cell wall thickening and stomatal development defect.

## Results

### ABI4 mutation rescued the lethal phenotype of flu

**Figure 1:**
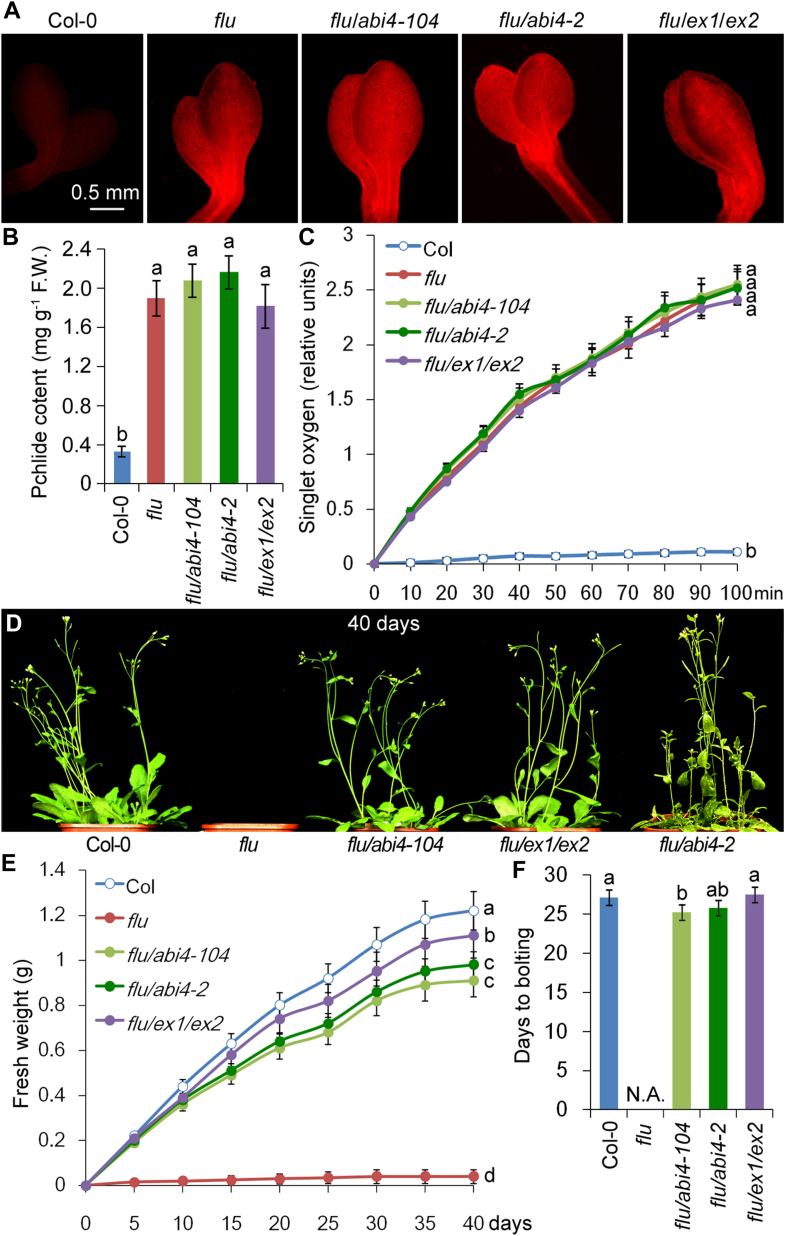
**Inactivation of either EXECUTERs or ABI4 results in *flu* mutant survival under light/dark cycles.***A*, fluorescence of 7-day-old WT (Col-0), *flu*, *flu*/*abi4* (*flu*/*abi4-104* and *flu*/*abi4-2*), and *flu*/*ex1*/*ex2* etiolated seedlings. *B*, Pchlide contents of 7-day-old etiolated seedlings. F.W., fresh weight. *C*, singlet oxygen in 7-day-old de-etiolated seedlings during 100 min of illumination (100 μmol photons m^−2^ s^−1^). *D*, forty-day-old seedlings grown under light/dark cycles. *E*, increasing fresh weight during 40 days of growth under light/dark cycles. *F*, flowering times of all four lines of plants grown under light/dark cycles. N.A., not available. Error bars show standard deviations (n = 3). Different lowercase letters indicate significant differences at the 0.05 (*p* < 0.05) level. ABI4, abscisic acid insensitive 4.

Two *abi4* mutants were used to perform genetic crossing with the *flu* mutant: *abi4-104* mutant (CS3839; single nucleotide substitution at codon 69 leading to missense E to K) and *abi4-2* mutant (SALK_080095; T-DNA insertion at codon 152) (13). In contrast to WT plants, all *flu* mutants (*flu*, *flu*/*abi4-2*, *flu*/*abi4-104,* and *flu*/*ex1*/*ex2*) accumulated five times higher levels of free Pchlide in the dark (Fig. 1, *A* and *B*). After transfer to the light, *flu*/*abi4-2*, *flu*/*abi4-104,* and *flu*/*ex1*/*ex2* generated singlet oxygen in amounts similar to that of *flu* (Fig. 1*C*). Despite their high Pchlide levels, both *flu*/*abi4-2* and *flu*/*abi4-104* rescued the lethal phenotype of *flu* (Fig. 1*D*). When grown under light/dark cycles, the *flu* seedlings ceased growth, whereas the *flu*/*abi4* and *flu*/*ex1*/*ex2* seedlings continued to grow at similar levels to the WT, except that their growth was slightly reduced and *flu*/*abi4* flowered a little earlier than the WT plants (Fig. 1, *E* and *F*). Under continuous light, all four lines grew equally well and finally reached the same flowering stage (Fig. S1).

It is interesting to note that Pchlide/^1^O_2_ accumulation levels in the dark were almost the same in the *flu* mutant, *flu*/*abi4* double-mutant, and *flu*/*ex1*/*ex2* triple-mutant (Fig. 1, *A*–*C*); however, only the *flu* mutant showed a lethal phenotype after illumination (Fig. 1*D*). This demonstrated that the growth inhibition and seedling lethality do not result from the physicochemical damage caused by ^1^O_2_ after the dark-to-light shift but are rather caused by the activation of a genetically determined stress response program (6, 7).

Rose Bengal (RB) functions as a photosensitizer by transferring energy to O_2_, generating ^1^O_2_ (14, 15). To confirm the specificity of RB action in nuclei, we grew 5-day-old *abi4-2*, *abi4-104*, and WT (Col-0) seedlings under 16-h light (100 μmol·m^−2^·s^−1^)/8-h dark cycles without RB for 5 days and then transferred to 1/2 Murashige and Skoog (MS) medium with 1, 10, or 100 μM RB for additional 6 days. Again, *abi4* mutants showed less photobleaching upon 100 μM RB treatment. However, all plants showed mild growth arrest at 10 μM RB treatment and severe growth arrest at 100 μM RB treatment may because of its toxicity at high concentrations (Fig. S2).

Both *35S:ABI4-GFP/flu/abi4-104* and *35S:ABI4-GFP/flu/abi4-2* complemented lines showed growth arrest and photobleaching under light/dark cycles (Fig. S3), confirming the irreplaceable role of ABI4 in ^1^O_2_ signaling. Given that both WT ABI4 protein and the single-point mutant ABI4 protein are expressed in the *35S-ABI4-GFP/flu/abi4-104* plants, they did not show the lethal phenotype of *flu*, but showed moderate growth arrest and photobleaching under light/dark cycles (Fig. S3). It could also be due to a dimer formation between the mutant ABI4 and WT ABI4-GFP, making a nonfunctional complex, or competition between them. To rule out the possibility that other proteins may interact with ABI4 and function in ^1^O_2_ signaling, the single-point mutant *abi4-104* with the full-length ABI4 ORF preserved was selected for the following experiments.

### Singlet oxygen signals regulate gene expression depending on ABI4

The binding strength of ABI4 with ^1^O_2_-responsive gene promoters was then evaluated using the chromatin immunoprecipitation (ChIP)-PCR method. For generating the antibody used for the ChIP assay, five epitopes of the ABI4 protein were synthesized chemically. The polyclonal antibodies against these epitopes were generated by inoculation in a mouse. The immune specificity of each antibody was verified by Western blotting (Fig. S4, *A* and *B*), and the antibody against epitope#11 was selected for the following experiments.

**Figure 2:**
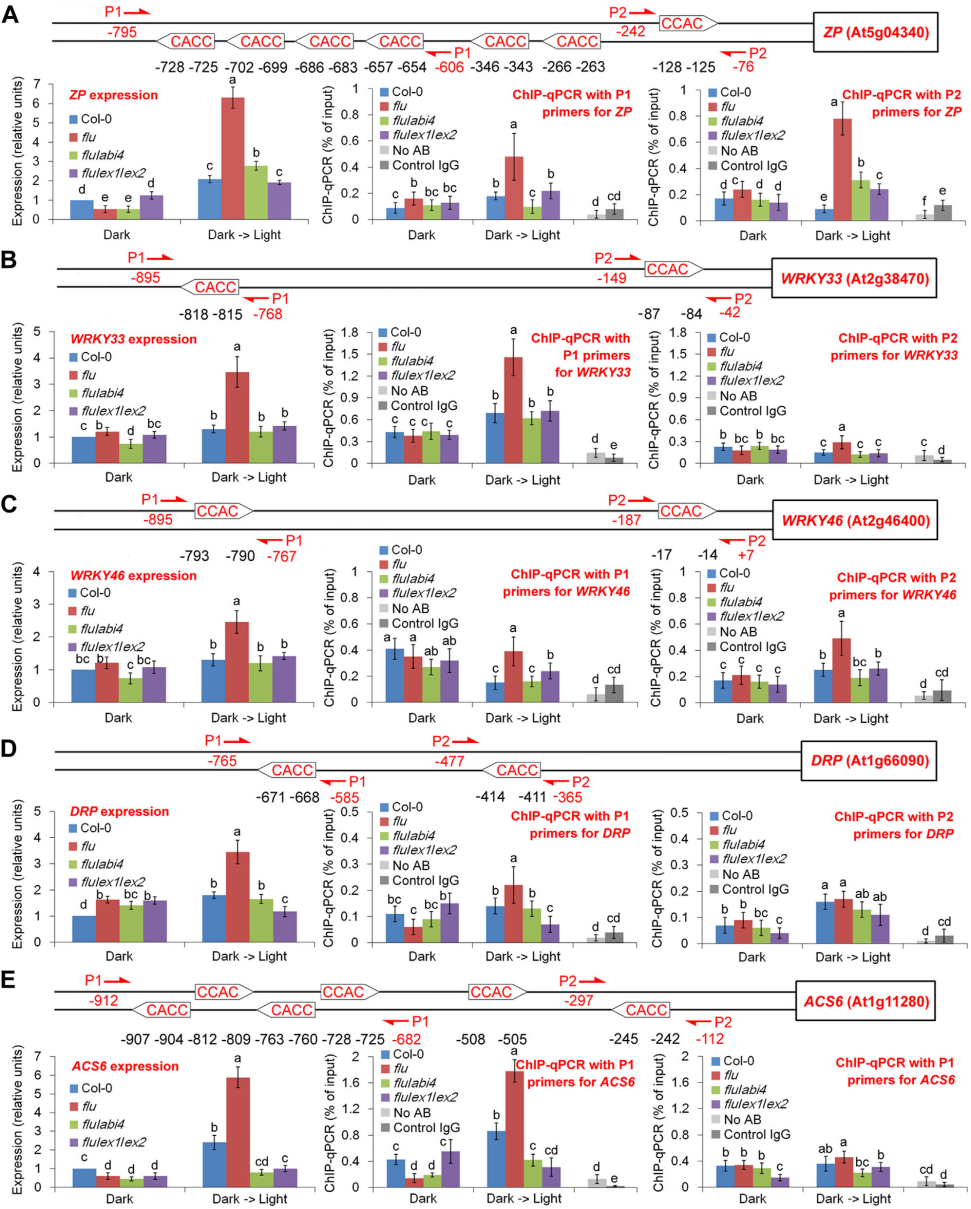
**Dark-to-light shift induces ABI4 binding to**^**1**^**O**_**2**_**-responsive gene promoters.** WT (Col-0), *flu*, *flu*/*abi4* (*flu*/*abi4-104*), and *flu*/*ex1*/*ex2* plants were grown for 21 days under continuous light, transferred to the dark for 8 h (dark), and in some cases reexposed to light for 30 min (dark -> light). Five representative genes, *ZP* (*A*), *WRKY33* (*B*), *WRKY46* (*C*), *DRP* (*D*), and *ACS6* (*E*) were studied. The positions of the CCAC motif and the corresponding ChIP-PCR primers (two pairs of primers were designed for each promoter: P1 and P2) are marked on the promoters. The binding strength of ABI4 with gene promoters was detected by ChIP-PCR. No AB shows the control signals in the “Dark -> Light” *flu* sample without the antibody (AB). Control IgG shows the control signals in the “dark -> light” *flu* sample with the mouse control IgG. Gene expression levels were detected by quantitative real-time PCR. The expression levels of the WT seedlings subjected to 8 h of dark were normalized to 100%. Error bars show standard deviations (n = 3). Different lowercase letters indicate significant differences at the 0.05 (*p* < 0.05) level. ABI4, abscisic acid insensitive 4; ACS6, 1-amino-cyclopropane-1 carboxylic acid (ACC) synthase 6; ChIP, chromatin immunoprecipitation; DRP, disease resistance protein; IgG, immunoglobulin IgG.

Previous studies indicated that the CCAC motif is a core element required for ABI4 binding (11, 16). This core binding element was found to be present at high frequencies in the promoters of five representative ^1^O_2_-inducible genes, including *ZP* (a putative C2H2 zinc finger transcription factor; At5g04340) (17), *WRKY33* (a transcription factor; At2g38470), *WRKY46* (a transcription factor; At2g46400), *DRP* (a disease resistance protein; At1g66090), and *ACS6* [1-amino-cyclopropane-1 carboxylic acid (ACC) synthase 6; At1g11280] (7). As shown in Figure 2, *A*–*E*, the dark-to-light shift significantly induced ABI4 binding to these gene promoters as well as their expression in the *flu* mutant, whereas these levels were only slightly increased or downregulated in *flu*/*abi4*, *flu*/*ex1*/*ex2,* or WT seedlings, also implying the indispensable roles of EXECUTER1/2 and ABI4 in ^1^O_2_ signaling. *WRKY46* primer pair 1 showed an unexpected pattern of high binding in the dark which was alleviated in the light (Fig. 2*C*). However, the fragment in *WRKY46* promoter contains both light-induced and light-repressed elements. Detailed regulatory mechanism needs further investigations.

Nevertheless, the induction rates of *ABI4* mRNA levels were much lower than the increasing rates of ABI4 binding (Fig. S5*A*), and no significant changes in ABI4 protein levels were observed after the dark-to-light shift in all four lines (Figs. S4*C* and S5*B*), indicating some other regulatory mechanism besides transcriptional or translational enhancement. Previous studies (11, 16) showed similar results whereby *ABI4* expression was barely affected by plastid signals. ABI4 might transmit the signals (bind to the target promoters) through a posttranslational regulation, such as phosphorylation or activity modulation through protein-protein interaction (18).

Recently, Li *et al.* (15) interestingly found that, upon light irradiation or RB treatment, EX1 transiently accumulated in the nucleus. Thus EX1 might interact with ABI4 to regulate ^1^O_2_-responsive genes. However, we failed to address a direct binding of EX1 to ABI4 by Coimmunoprecipitation (Co-IP) experiments (Fig. S6). Whether ABI4 is a direct mediator of the signal or a downstream target of EX1 in the signaling network requires further studies.

**Figure 3:**
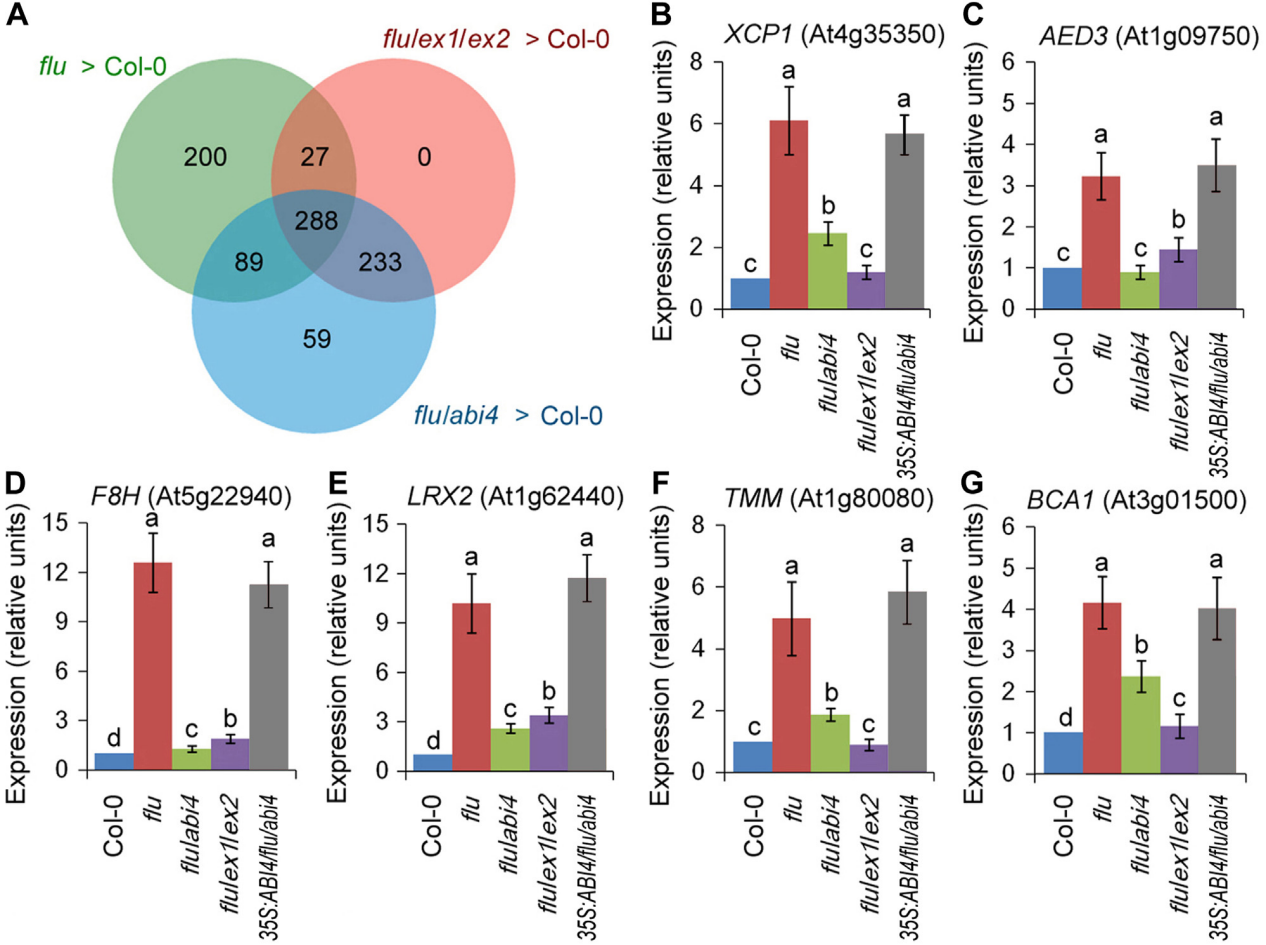
^**1**^**O**_**2**_**-induced genes in *flu* mutant specifically.** WT (Col-0), *flu*, *flu*/*abi4* (*flu*/*abi4-104*), *flu*/*ex1*/*ex2* and *35S:ABI4-GFP/flu/abi4-2* plants were grown for 21 days under continuous light, transferred to the dark for 8 h, and then reexposed to light for 30 min. *A*, The relationships of three selected groups of genes that were upregulated by at least 2-fold in *flu versus* the WT, *flu*/*abi4 versus* the WT, and *flu*/*ex1*/*ex2 versus* the WT were analyzed using a Venn diagram. *B*–*G*, the expression levels of six representative genes were detected by quantitative real-time PCR: two cell-death genes *XCP1* (*B*) and *AED3* (*C*), two cell wall organization genes *F8H* (*D*) and *LRX2* (*E*), and two stomatal development genes *TMM* (*F*) and *BCA1* (*G*). The expression levels of the WT seedlings were normalized to 100%. Error bars show standard deviations (n = 3). Different lowercase letters indicate significant differences at the 0.05 (*p* < 0.05) level. AED3, apoplastic EDS1 (enhanced disease susceptibility 1)-dependent 3; BCA1, beta carbonic anhydrase 1; F8H, fragile fiber 8 homolog; LRX2, leucine-rich repeat/extensin 2; TMM, too many mouths; XCP1, xylem cysteine peptidase 1.

The ^1^O_2_-dependent nuclear gene expression changes were clarified by RNA-seq [the principal component analysis is shown in Fig. S7; heatmaps of differentially expressed genes (DEGs) are shown in Fig. S8]. Genes with a 2-fold or greater transcript level than the control were considered to be significantly upregulated. After 30 min of reillumination, a total of 896 genes had been upregulated in *flu* or *flu*/*abi4* or *flu*/*ex1*/*ex2* relative to the WT (Fig. 3*A*). Among them, 200 transcripts were ^1^O_2_-induced genes in *flu* specifically, including 30 genes regulating cell wall organization (29 genes) or lignin catabolic process (1 gene), 22 phytohormone-regulated genes (six cytokinin-regulated genes; five abscisic-acid-regulated genes; four gibberellin-regulated genes; three brassinosteroid-regulated genes; one ethylene-regulated gene; one auxin-regulated gene; one salicylic-acid-regulated gene; one jasmonic-acid-regulated gene), 20 stress-responsive genes (11 genes responsive to water deprivation; five genes responsive to cold; two genes responsive to salt stress; two genes responsive to oxidative stress), 15 photosynthesis-related genes, 11 lipid metabolic process genes, two genes related to stomatal development, and two genes related to cell death (Data sheet S1). We also analyze the DEGs between *flu* and *flu*/*abi4*. Among them, top ten DEGS with the biggest differences included three cell wall organization genes, one ethylene-responsive gene, one stress-responsive gene and one cell death-related gene (Data sheet S1). Gene Ontology (GO) enrichment analysis also showed overrepresented GO terms related to cell wall organization, pectin biosynthesis, and salicylic acid binding (Fig. S9). A previous study also indicated that genes in jasmonic acid signaling pathway were induced by the application of RB in the light or dark-to-light transitions in the *flu* mutant independently of the hormone (14).

Interestingly, 95% (521/548) of upregulated genes in *flu*/*ex1*/*ex2* relative to the WT were also upregulated in *flu*/*abi4* relative to the WT (Fig. 3*A*), suggesting that ABI4 functions downstream of EXECUTER1/2. The expression levels of six representative genes were detected by quantitative real-time PCR, including two cell-death genes *XCP1* (xylem cysteine peptidase 1; At4g35350) (19) and *AED3* [apoplastic EDS1 (enhanced disease susceptibility 1)-dependent 3; At1g09750] (20), two cell wall organization genes *F8H* (fragile fiber 8 HOMOLOG; At5g22940) (21) and *LRX2* (leucine-rich repeat/extensin 2; At1g62440) (22), and two stomatal development genes *TMM* (too many mouths; At1g80080) (23) and beta carbonic anhydrase 1(*BCA1*); At3g01500) (24). These genes were all induced more than three times in *flu* mutant or *35S:ABI4-GFP/flu/abi4-2* complemented plants after the dark-to-light shift but were nonsignificantly or less induced in the WT, *flu*/*abi4* or *flu*/*ex1*/*ex2* seedlings (Fig. 3, *B*–*G*), further confirming the key roles of EXECUTER1/2 and ABI4 in ^1^O_2_ signaling.

**Figure 4:**
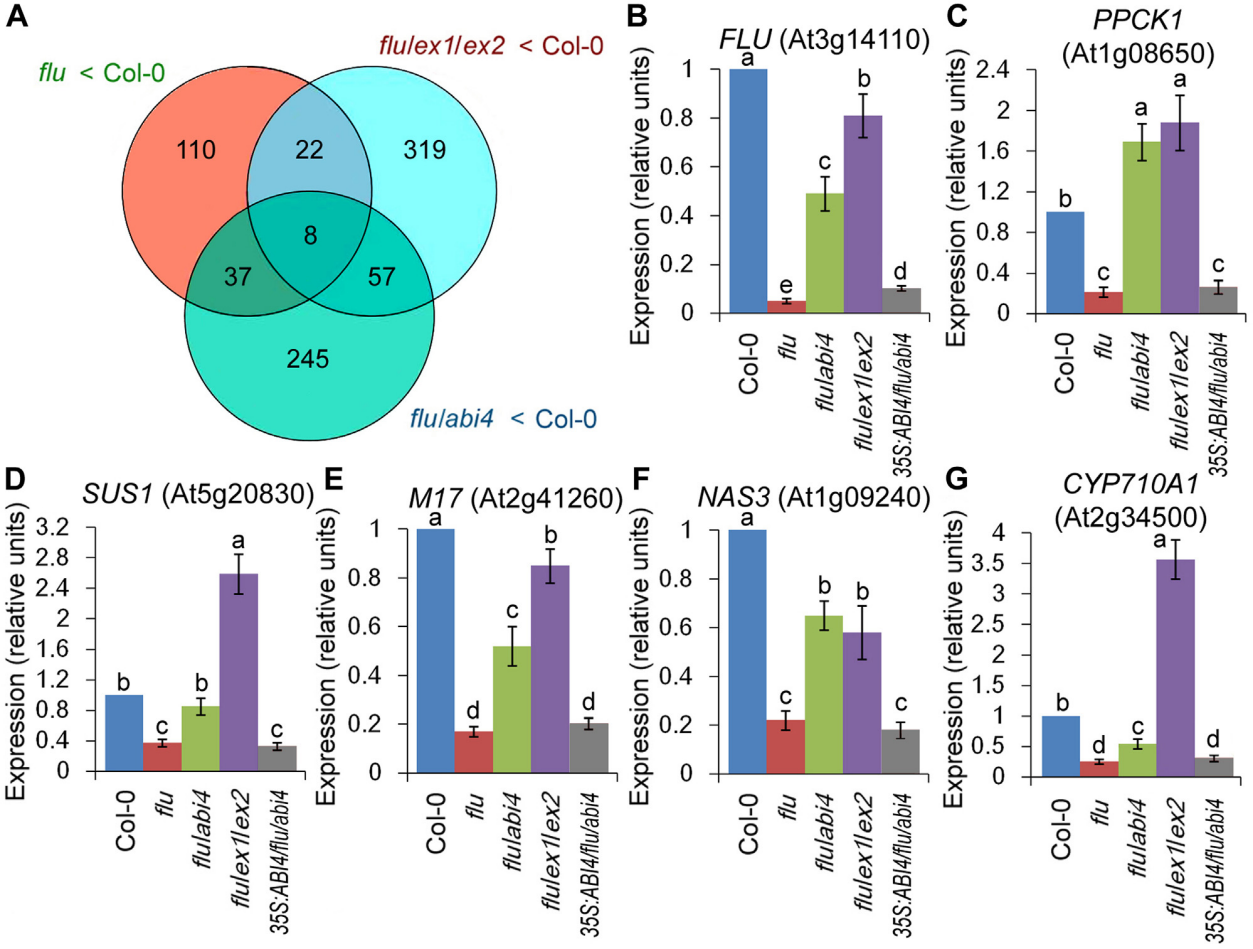
^**1**^**O**_**2**_**-repressed genes in *flu* mutant specifically.** WT (Col-0), *flu*, *flu*/*abi4* (*flu*/*abi4-104*), *flu*/*ex1*/*ex2* and *35S:ABI4-GFP/flu/abi4-2* plants were grown for 21 days under continuous light, transferred to the dark for 8 h, and then reexposed to light for 30 min. *A*, the relationships of three selected groups of genes that were downregulated by at least 2-fold in *flu versus* the WT, *flu*/*abi4 versus* the WT, and *flu*/*ex1*/*ex2 versus* the WT were analyzed using a Venn diagram. *B*–*G*, the expression levels of six representative genes were detected by quantitative real-time PCR: FLU (*B*), *PPCK1* (*C*), *SUS1* (*D*), and *M17* (*E*), *NAS3* (*F*) and *CYP710A1* (*G*). The expression levels of the WT seedlings were normalized to 100%. Error bars show standard deviations (n = 3). Different lowercase letters indicate significant differences at the 0.05 (*p* < 0.05) level. ABI4, abscisic acid insensitive 4; CYP, cytochrome P450; NAS3, nicotianamine synthase 3; PPCK1, phosphoenolpyruvate carboxylase kinase; SUS1, SUS1, sucrose synthase 1.

^1^O_2_-repressed genes were also analyzed. After 30 min of reillumination, a total of 798 genes had been downregulated in *flu* or *flu*/*abi4* or *flu*/*ex1*/*ex2* relative to the WT (Fig. 4*A*). Among them, 110 transcripts were ^1^O_2_-repressed genes in *flu* specifically, including seven genes encoding lipid metabolic or lipid-binding proteins, seven genes responsive to water deprivation, six genes regulating pollen development or pollen tube growth (including two nicotianamine synthase genes), five genes controlling carbohydrate metabolism or transport, four genes related to seed dormancy or development and four cytochrome p450 genes (Data sheet S2). We also analyze the DEGs between *flu* and *flu*/*abi4*. Among them, top ten DEGS with the biggest differences included three lipid (sterol) metabolic genes, two phenylpropanoid biosynthetic (water deprivation-responsive) gene and one chlorophyll biosynthetic gene (Data sheet S2). GO enrichment analysis also showed overrepresented GO terms related to sterol biosynthetic process, water deprivation, and nicotianamine biosynthesis (Fig. S10).

Interestingly, *FLU* gene was 20-fold repressed by the ^1^O_2_ signal (Fig. 4*B*). The expression levels of other five representative genes were detected by quantitative real-time PCR, including two carbohydrate metabolism genes *PPCK1* (encoding a phosphoenolpyruvate carboxylase kinase; At1g08650) (25) and *SUS1* (encoding a water-deprivation-responsive sucrose synthase; At5g20830) (26), one seed development genes *M17* (encoding a late embryosis abundant protein; At2g41260) (27), one pollen development gene *NAS3* (encoding a nicotianamine synthase; At1g09240) (28) and a sterol metabolic cytochrome (CYP) P450 gene *CYP710A1* (At2g34500) (29). These genes were all reduced more than 2.5 times in *flu* mutant or *35S:ABI4-GFP/flu/abi4-2* complemented plants after the dark-to-light shift but were nonsignificantly or less reduced in the WT, *flu*/*abi4* or *flu*/*ex1*/*ex2* seedlings (Fig. 4, *C*–*G*), further confirming the key roles of EXECUTER1/2 and ABI4 in ^1^O_2_ signaling.

### Singlet oxygen signals induce stomatal development defect and cell wall thickening under light/dark cycles

Consistent with the RNA-seq data, under the condition of light/dark cycles, abrupt ROS accumulation, lipid peroxidation, and cell death were observed in the *flu* mutant, but not in the *flu*/*abi4* or *flu*/*ex1*/*ex2* mutants (Fig. S11). Although all *flu* mutants accumulated the same high level of Pchlide in the dark (Fig. 1, *A* and *B*), the Pchlide was converted into chlorophylls in *flu*/*abi4* and *flu*/*ex1*/*ex2* under light, and thus their Pchlide declined to low levels similar to that of the WT seedlings (Fig. S12, *B* and *C*). By contrast, although a certain level of chlorophylls (about one-fourth of the WT; Fig. S12*F*) was synthesized in the *flu* mutant grown under 7-days light/dark cycles and then transferred to reillumination for 1 h, the plants became bleached (the steady-state chlorophyll level declined to 1/25 of the WT; Fig. S12*E*), and their Pchlide could not be converted into chlorophylls and therefore accumulated (Fig. S12*C*). Abrupt ROS accumulation (Fig. S11) and dramatically declined *FLU* gene expression (Fig. 4*B*) in *flu* mutant after dark-to-light shifts may be two of the reasons.

**Figure 5:**
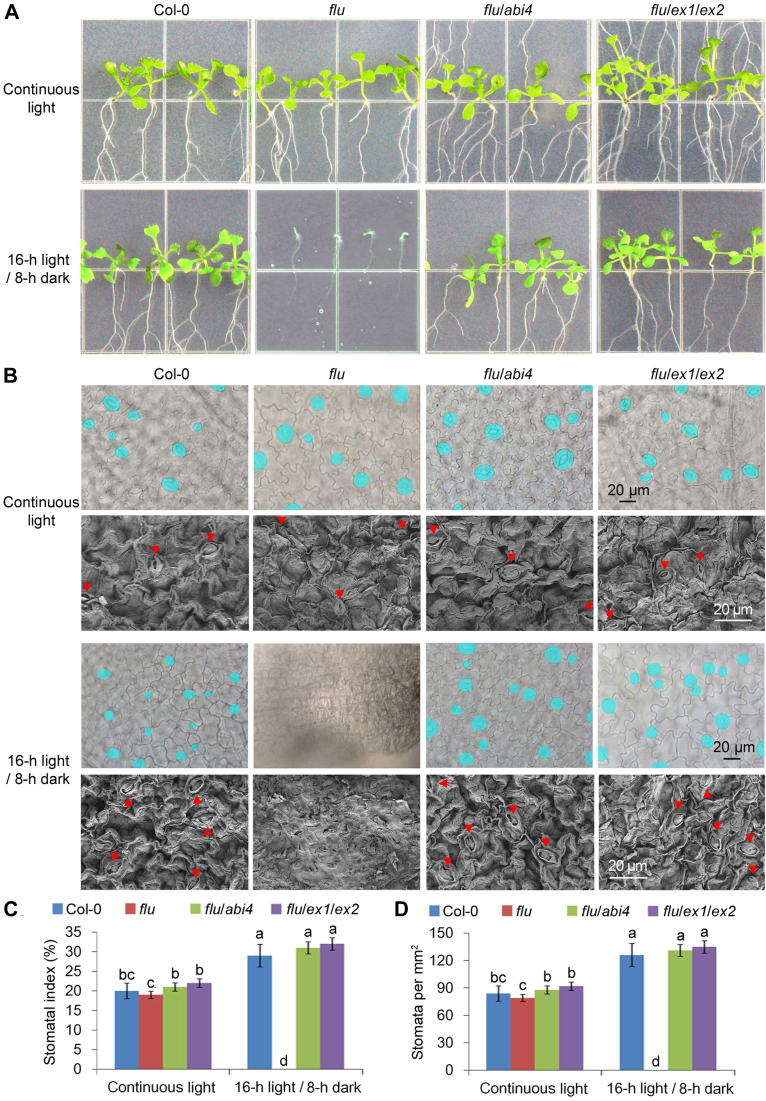
^**1**^**O**_**2**_**-signal induces stomatal development defect under light/dark cycles.** Cotyledons from 11-day-old WT (Col-0), *flu*, *flu*/*abi4* (*flu*/*abi4-104*) and *flu*/*ex1*/*ex2* seedlings grown under light/dark cycles or continuous light were collected (*A*). The cotyledons were fixed and observed with a light microscope. Stomata are false colored in *blue* for easier identification (*B*). And the cotyledons were also fixed and observed with a scanning microscope. *Red arrows* indicate the stomata (*B*). Stomatal index (*C*) and stomatal density (*D*) were counted. Error bars show standard deviations (n = 5). Different lowercase letters indicate significant differences at 0.05 (*p* < 0.05) levels.

**Figure 6:**
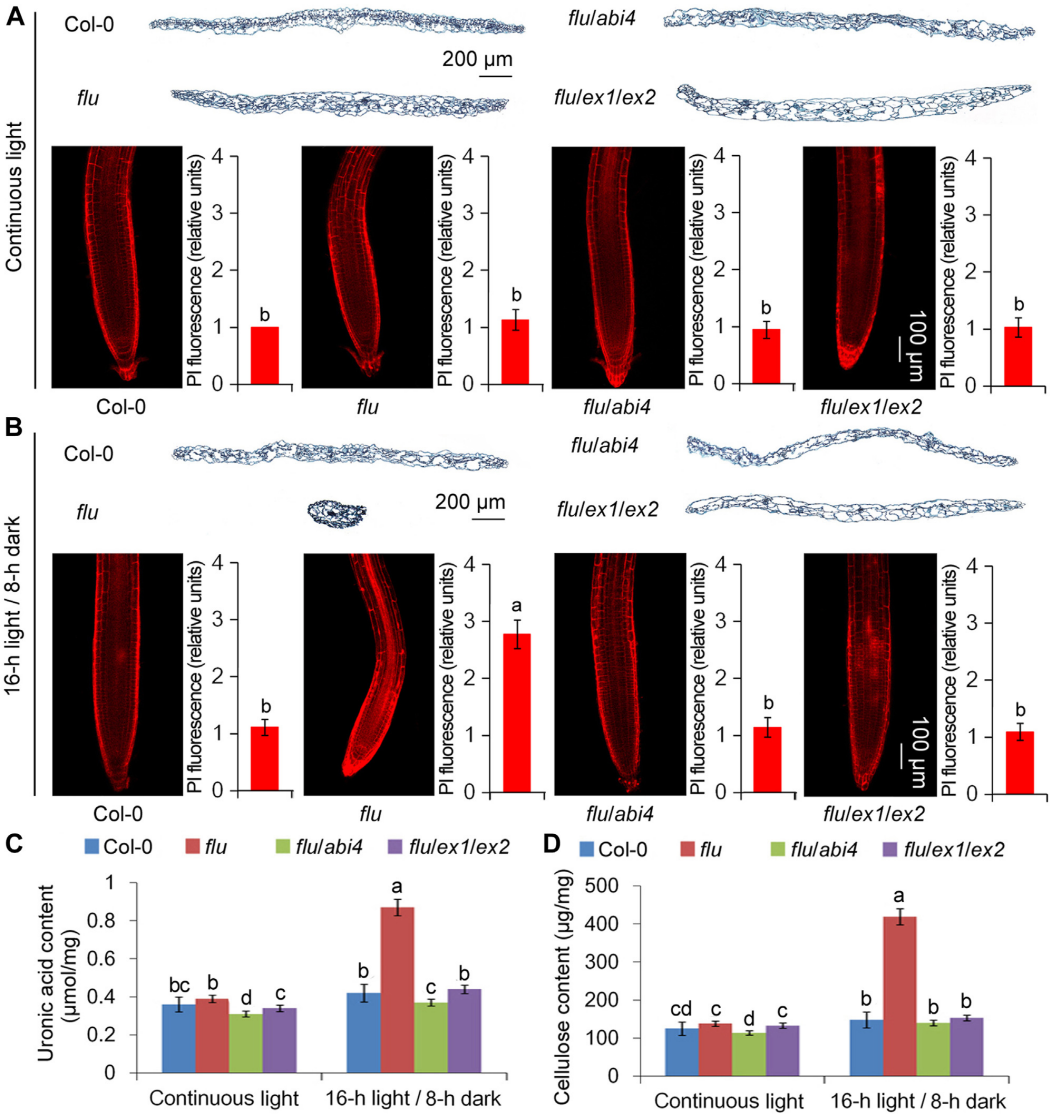
^**1**^**O**_**2**_**-signal induces cell wall thickening under light/dark cycles.** Cotyledons from 11-day-old WT (Col-0), *flu*, *flu*/*abi4* (*flu*/*abi4-104*) and *flu*/*ex1*/*ex2* seedlings grown under continuous light (*A*) or light/dark cycles (*B*) were collected. The paraffin cross sections of cotyledons were made by staining with safranine and solid green (*A* and *B*). Cell walls of the roots were stained with propidium iodide (PI). And the PI fluorescence signals were quantified and shown in the *right panel* (*A* and *B*). Uronic acid content (*C*) and cellulose content (*D*) of cell walls were detected. F.W., fresh weight. Error bars show standard deviations (n = 5). Different lowercase letters indicate significant differences at 0.05 (*p* < 0.05) levels.

Besides oxidative damage, interestingly, no stomata have been observed on the *flu* cotyledons grown under light/dark cycles (Fig. 5, *B*–*D*). Furthermore, apparent cell wall thickening (by quantifying propidium iodide fluorescence signals; Fig. 6, *A* and *B*) and increases in pectin (uronic acid) and cellulose contents of cell walls (Fig. 6, *C* and *D*) were also observed in the *flu* mutant grown under light/dark cycles, but not in the other lines. As an integrated effect of stomatal development defect and cell wall thickening, following the dark-to-light shifts, *flu* stopped growing, whereas *flu*/*abi4* and *flu*/*ex1*/*ex2* grew normally.

### Moderate singlet oxygen signals enhance plant tolerance to environmental stresses

**Figure 7:**
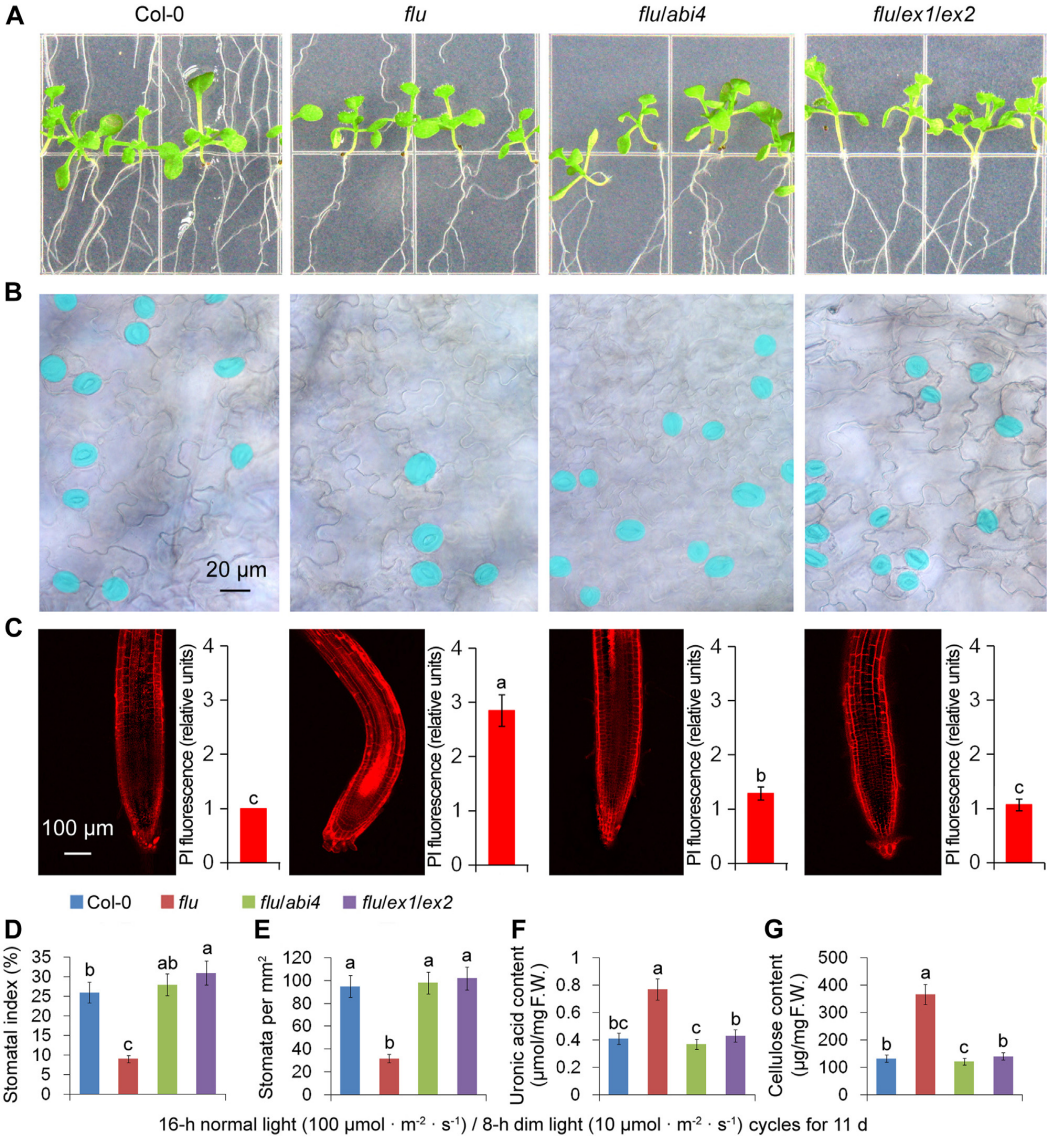
^**1**^**O**_**2**_**-signal induces cell wall thickening with much less stomata in the *flu* mutant under normal light/dim light cycles.** Seedlings of 11-day-old WT (Col-0), *flu*, *flu*/*abi4* (*flu*/*abi4-104*) and *flu*/*ex1*/*ex2* seedlings grown under 16-h normal light (100 μmol·m^−2^·s^−1^)/8-h dim light (10 μmol·m^−2^·s^−1^) cycles (*A*) were collected. The cotyledons were fixed and observed with a light microscope. Stomata (*B*) are false colored in *blue* for easier identification. Cell walls of the roots were stained with propidium iodide (PI). And the PI fluorescence signals were quantified and shown in the *right panel* (*C*). Stomatal index (*D*) and stomatal density (*E*) were counted. Uronic acid content (*F*) and cellulose content (*G*) of cell walls were detected. F.W., fresh weight. Error bars show standard deviations (n = 5). Different lowercase letters indicate significant differences at 0.05 (*p* < 0.05) levels.

When culturing, we noticed that, if the complete darkness was replaced by a dim light, the *flu* mutant could grow up. Although about 50% higher Pchlide (Figs. S13*A*) and 120% higher ^1^O_2_ (Fig. S13*B*) accumulated, no apparent growth arrest was observed for the *flu* mutant under 16-h normal light (100 μmol·m^−2^·s^−1^)/8-h dim light (10 μmol·m^−2^·s^−1^) cycles (Fig. 7*A* and Fig. S13, *C*–*E*). Nevertheless, stomatal density reduction (by two-thirds; Fig. 7, *B*, *D*, and *E*) and cell wall thickening (doubled; Fig. 7, *C*, *F*, and *G*) and still occurred under normal light/dim light cycles.

**Figure 8:**
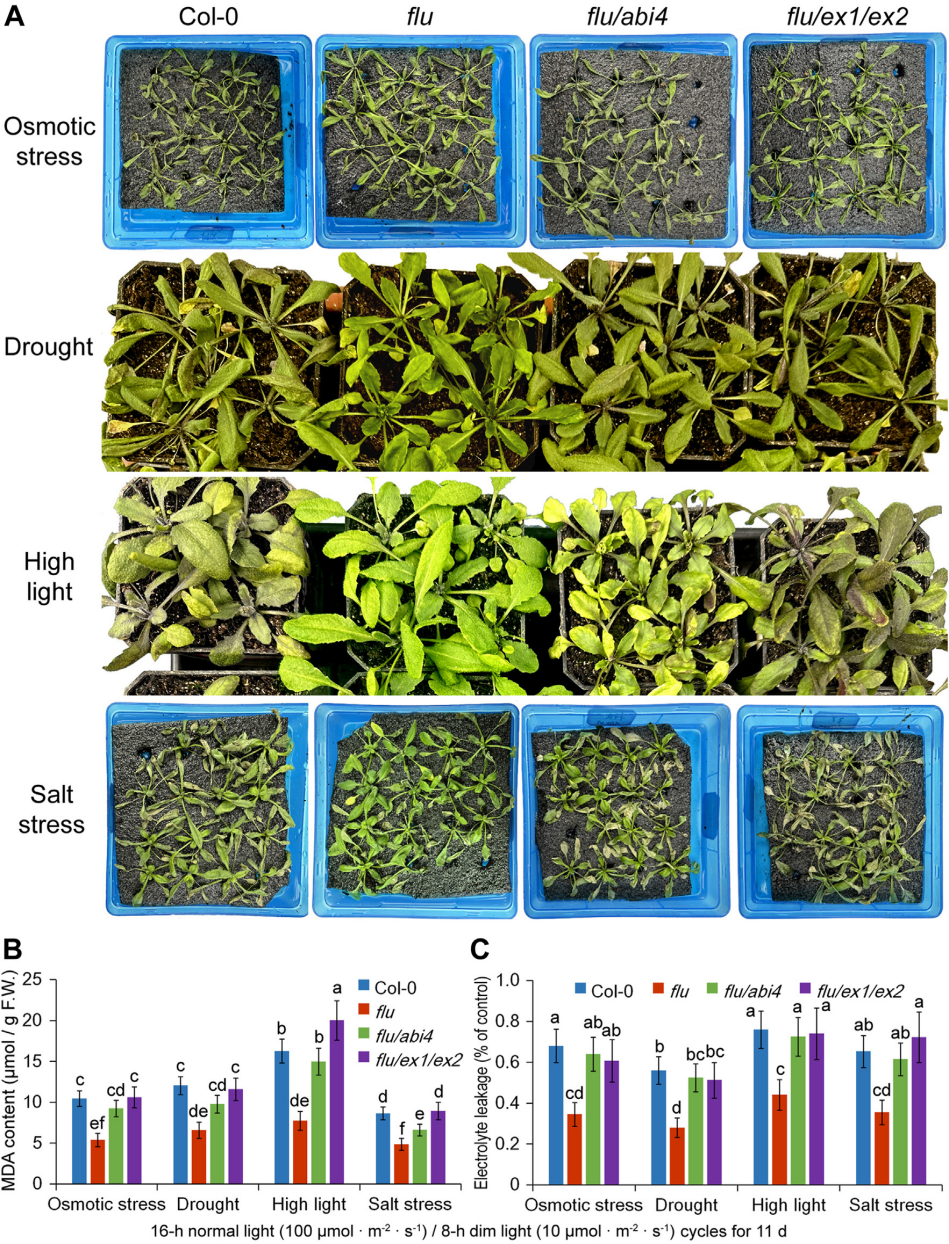
**The *flu* mutant shows greater tolerance to environmental stresses under normal light/dim light cycles.** Seedlings of 21-day-old WT (Col-0), *flu*, *flu*/*abi4* (*flu*/*abi4-104*) and *flu*/*ex1*/*ex2* seedlings grown under 16-h normal light (100 μmol·m^−2^·s^−1^)/8-h dim light (10 μmol·m^−2^·s^−1^) cycles were subjected to environmental stresses (*A*). After 3-days osmotic stress with 16% PEG 6000 solution, 14-days drought treatment (withholding water), 3-h high-light stress (1500 μmol of photon m^−2^ s^−1^) or 3-days saline treatment (100 mM NaCl), malondialdehyde (MDA) contents (*B*) and electrolyte leakage (*C*) were measured. F.W., fresh weight. Error bars show standard deviations (n = 5). Different lowercase letters indicate significant differences at 0.05 (*p* < 0.05) levels.

The stress tolerance of ^1^O_2_-signaling mutants has been investigated further. The plants were grown under 16-h normal light (100 μmol·m^−2^·s^−1^)/8-h dim light (10 μmol·m^−2^·s^−1^) cycles for 21 days, and then subjected to environmental stresses. After 3-days osmotic stress with 16% PEG 6000 solution, 14-days drought treatment (withholding water), 3-h high-light stress (1500 μmol of photon m^−2^ s^−1^) or 3-days saline treatment (100 mM NaCl), malondialdehyde contents and electrolyte leakage (EL) increased dramatically in WT seedlings and *flu*/*abi4* and *flu*/*ex1*/*ex2* mutants (Fig. 8). On the contrary, the *flu* mutant showed a greater resistance to all the four types of stresses (Fig. 8). All the data suggested that moderate ^1^O_2_-signals (induced by normal light/dim light cycles) play a positive role in plant’s adaptation to environmental stresses.

## Discussion

A recent study indicated that EX1 protein undergoes tryptophan (Trp) 643 oxidation by singlet oxygen (30), leading to the generation of oxidized Trp variants and priming EX1 degradation *via* a membrane-bound FtsH protease (9). While EX2 also encounters ^1^O_2_-dependent Trp530 oxidation and FtsH-dependent turnover, and functions as a negative regulator of the EX1 (30). Future studies should clarify how the ^1^O_2_ signals are transducted. All ^1^O_2_-signaling factors identified previously (FLU, EX1, and EX2) were plastid proteins, but how the ^1^O_2_-signals are transferred from the plastids into the nucleus is still unknown. Some plastid proteins may be secreted and thus a part of the signaling network, such as chloroplast preprotein and amino acid transporters (31, 32).

Alternatively, upon induction by ^1^O_2_, EX1 may transiently accumulate and translocate from plastids to the nucleus. Then nuclear EX1 may act as a transcriptional coactivator and interact with the transcription factors WRKY18 and WRKY40 to promote the expression of ^1^O_2_-responsive genes (15). In this study, we found that the transcription factor ABI4 may also be involved in the ^1^O_2_-signaling transcriptional network, although EX1 may not bind to ABI4 directly. It has been reported that WRKY18, WRKY40, and WRKY60 interacted with the W-box in the promoters of *ABI4* and *ABI5* genes (33). Therefore, EX1 might modulate the ^1^O_2_ signaling pathway by interacting with multiple transcription factors (*e.g.*, WRKY18 and WRKY40) and then operate *ABI4* gene indirectly, which requires further studies.

^1^O_2_-induced genes include 20 stress-responsive genes, 30 cell wall biosynthesis or organization genes, and two genes related to stomatal development (Data sheet S1). Exponentially upregulation of two negative stomatal development regulators TMM and BCA1 by ^1^O_2_ signals (Fig. 3, *F* and *G*) might be associated with the stomatal development defect in the *flu* cotyledons. However, the stomatal development phenotype of *flu* mutant could not be only attributed to higher expression of *TMM* and *BCA1*. Cell wall organization also regulates stomatal development. For example, pectin methylesterification in guard cell walls plays a key role in stomatal dynamics and stomatal response to external stimuli (34). The Arabidopsis sensitive-to-freezing 8 (*sfr8*) mutant exhibits reduced cell wall fucose levels and larger stomatal pore complexes, and its guard cell lacks a fully developed cuticular ledge. Fucosylation-dependent dimerization of the cell wall pectic domain rhamnogalacturonan-II may be essential for normal cuticular ledge development (35). Detailed cell wall component/structure changes and stomatal development regulations trigged by ^1^O_2_ signals need further explorations.

A large number of ^1^O_2_-repressed genes have also been identified in the *flu* mutant specifically (Data sheet S2). Given that genes related to pollen and seed development were significantly suppressed in the *flu* mutant, the regulation to plant reproductive growth by singlet oxygen signaling would be an interesting future research direction.

The phenotype of stomatal density reduction and cell wall thickening induced by singlet oxygen has important implications for agricultural industry. Declines in stomatal density would enhance plant's tolerance to drought (36, 37, 38, 39, 40), salinity/osmotic stress (38, 39, 40, 41, 42), and heat stress (40, 43, 44) by reducing water transpiration. There are interests in developing crop varieties that require less water yet still maintain the grain yield. This requirement may be fulfilled by altering stomatal development that the crop varieties with fewer stomata may be more conservative in their water use and therefore more tolerant to drought, salinity, or heat stress (45). Plant cell walls are the first physical barrier against pathogenic invasion. Under biotic stresses, plants thicken their cell walls to strengthen them and restrain pathogenic infections (46, 47, 48). And cell wall thickening would enhance plant's tolerance to drought (48, 49), salinity/osmotic stress (50, 51), metal stress (52, 53), and especially waterlogging stress (49, 54, 55) by improving mechanical strength and facilitating water transport. Tailoring the content and structure of plant cell walls could modulate growth, abiotic/biotic stress resistance, biomass yield, and other important agronomic traits (55). Illuminated by this study, all these goals of cell wall thickening and stomatal reduction may be simply achieved by knocking out the *FLU* gene, accompanying with appropriate light cycles (periodic alternation of light intensity). Future experiments with *FLU*-null plants are warranted.

## Experimental procedures

### Plant materials and growth conditions

Arabidopsis mutants were in the Col-0 background. The *flu* mutant and *flu,ex1,ex2* triple mutant were provided by Prof. Chanhong Kim (Chinese Academy of Sciences, China). Seeds of *35S:EX1-GFP* transgenic plants were a gift from Prof. Rongcheng Lin (Institute of Botany, Chinese Academy of Sciences). Seeds of *abi4-102* mutant (CS3837; W to STOP at codon 80), *abi4-104* mutant (CS3839; single nucleotide substitution at codon 69 leading to missense E to K) and *abi4-2* mutant (SALK_080095; T-DNA insertion at condon 152) were obtained from the Arabidopsis Biological Resource Center at Ohio State University (Columbus, OH, USA). *flu,abi4-2* double mutant and *flu,abi4-104* double mutant were generated by performing genetic crossing respectively, and the progenies were analyzed by PCR. The *35S:ABI4-GFP* construct (56) was crossed into either *flu,abi4-2* or *flu,abi4-104* double mutant background. After vernalization at 4 °C for 3 days, the seeds were sterilized in 75% (v/v) alcohol and 0.1% (v/v) HgCl_2_, and then grown on 1/2 MS medium with 1% sucrose or on soils under continuous light (100 μmol·m^−2^·s^−1^), 16-h light (100 μmol·m^−2^·s^−1^)/8-h dark cycles or 16-h normal light (100 μmol·m^−2^·s^−1^)/8-h dim light (10 μmol·m^−2^·s^−1^) cycles at 21 °C ± 1 deg. C.

### Stress treatments

WT plants (Col-0), *flu* mutant, *flu,abi4-104* double mutant, and *flu,ex1,ex2* triple mutant were grown under 16-h normal light (100 μmol·m^−2^·s^−1^)/8-h dim light (10 μmol·m^−2^·s^−1^) cycles for 21 days, and then subjected to four types of environmental stresses. Osmotic stress was imposed by submerging roots into half strength Hoagland solution containing 16% (w/v) PEG-6000 with an osmotic potential of −0.5 MPa for 3 days. Drought treatment was performed by withholding water for 14 days. For high-light stress, the seedlings were exposed to 1500 μmol of photon m^−2^ s^−1^ for 3 h. For salt stress, plants were treated with 100 mM NaCl for 3 days.

### Analysis of flowering time

For seedlings grown in 1/2 MS media, we scored the number of rosette leaves at the stage of bolting when stems were about 3 mm (57). We also scored days to bolting. 20 rosettes for each sample were counted. The experiments were repeated three times.

### Measurement and visualization of protochlorophyllide

Protochlorophyllide (Pchlide) were extracted from 7-days etiolated seedlings (0.2 g seedlings to 0.5 ml alkaline acetone) as described by Lay *et al.* (58). Etiolated cotyledons of 5-day-old seedlings were exposed to blue light and the emitted Pchlide fluorescence was recorded using the Leica M165C/FC fluorescent microscope (Leica Microsystems) (9).

Pchlide and chlorophylls were also extracted with 80% acetone from the seedlings grown under continuous light, 16 h/8 h light/dark cycles or 16-h normal light/8-h dim light cycles for 7 days (at the time-points of 0-h and 1-h illumination after the seventh cycle). Pchlide was quantified by using the standard chemical, which was purchased from Frontier Scientific (Logan Comp). Chlorophyll (Chl) content was measured according to the method of Lichtenthaler and Wellburn (59).

### ROS visualization and cell-death staining

Superoxide and H_2_O_2_ levels were visually detected with nitro blue tetrazolium and 3,3-diaminobenzidine, respectively, as described previously (60). Seven-day-old seedlings were excised at the base with a razor blade and supplied through the cut ends with nitro blue tetrazolium (0.5 mg ml^−1^) or 3,3-diaminobenzidine (2 mg ml^−1^) solutions for 8 h. Leaves were then decolorized in boiling ethanol (95%) for 20 min. At least three leaves were used for each treatment.

The singlet oxygen level was determined with the fluorescence probe Singlet Oxygen Sensor Green Reagent (Molecular Probes Inc). Singlet oxygen generation ratios were determined for 7-day-old etiolated seedlings after transfer to normal light (100 μmol photons m^−2^ s^−1^) (61), or 7-day-old seedlings grown under 16-h normal light/8-h dim light cycles and then transferred to normal light for 1 h.

The photobleaching (cell death) was visually detected by trypan-blue staining (1.25 mg mL^−1^) (62).

### Measurements of malonaldehyde and electrolyte leakage

The extraction of malondialdehyde in 7-day-old seedlings was performed using thiobarbituric acid solution according to the method of Chen *et al.* (63) After centrifugation, the absorbance of the supernatant was monitored at 532 nm and corrected for nonspecific turbidity by subtracting the absorbance at 600 nm. EL was determined by using a conductivity meter (DDSJ-308A, Shanghai Precision Instruments Co Ltd) according to the method of Chen *et al.* (63) The relative EL was obtained according to the ratio of the initial conductivity to the absolute conductivity.

### Pectin and cellulose content determination

Extraction of cell wall components from 11-days-old seedlings grown under continuous light, 16 h/8 h light/dark cycles or 16-h normal light/8-h dim light cycles was carried out according to Xiao *et al.* (64) and Du *et al.* (65). Pectin was extracted from the cell wall extract by boiling 2 mg of the extract with 1 ml ultrapure water three times, 1 h each. Each boiling was followed by centrifugation for 3 min at 4500*g* and collection of the supernatant into one tube. Uronic acid content was measured as described by Xiao *et al.* (64) and Du *et al.* (65), using galacturonic acid as reference. Crystalline cellulose content was measured following a procedure described by Xiao *et al.* (64) and Du *et al.* (65).

### Microscopy analysis of root and cotyledon cell walls

To visualize root cell walls, roots of 11-day-old seedlings grown under continuous light, 16 h/8 h light/dark cycles or 16-h normal light/8-h dim light cycles were stained with 10 μg/ml propidium iodide for 20 min at room temperature (66). Signals of the stained roots were then excited by light with wave length of 595 nm and imaged under an Olympus FluoView FV1000 confocal microscope.

To visualize cotyledon cell walls, the paraffin cross sections of cotyledons were made by staining with safranine and solid green. The slices were scanned and imaged under the stereo microscope (Leica Microsystems M165C/FC).

### Stomatal count

Seedlings of 11-day-old seedlings grown under continuous light or under continuous light, 16 h/8 h light/dark cycles or 16-h normal light/8-h dim light cycles were collected in 70% ethanol, cleared overnight at room temperature, and then stored in Hoyer’s solution and observed with a light microscope (Leica Microsystems M165C). Stomatal density is the number of stomata per unit of area. To count the stomatal density, five square areas of 0.5 mm^2^ adaxial epidermises were examined for each cotyledon, and the amounts were averaged to yield a predicted stomatal density. Cotyledons from at least eight different plants were selected for all genotypes. The stomatal index was calculated using the following formula: stomatal index = (number of stomata)/(total epidermal cells) × 100% (67).

A scanning microscope (GeminiSEM300, Carl Zeiss) was also applied to determine the surface characteristics of adaxial epidermises and stomata of cotyledons. The samples were prefixed overnight at 4 °C in 3% glutaraldehyde and 0.1 M sodium cacodylate buffer (pH 6.9).

### RNA-seq

WT (Col-0), *flu*, flu,abi4, and *flu,ex1,ex2* mutant plants were grown for 21 days under continuous light, shifted to the dark for 8 h, and reexposed to light for 30 min. Equal quantities of RNA from three independent biological replicates of each plant were pooled for complementary DNA (cDNA) library construction. Oligo-(dT) magnetic beads were used to isolate poly-(A) mRNA from total RNA, and the mRNA was fragmented in a fragmentation buffer. Using these short fragments (about 200 bp in length) as templates, random hexamer-primers were used in first-strand cDNA synthesis. Second-strand cDNA was synthesized using the appropriate buffer, dNTPs, RNaseH, and DNA polymerase I. Short double-stranded cDNA fragments were purified with a Qiaquick PCR extraction kit (Qiagen), resolved with an buffer EB (Qiagen) for end-reparation and for adding poly (A), and then ligated to sequencing adapters. After purification *via* agarose gel electrophoresis, suitable fragments were enriched by PCR amplification. Finally, the libraries were sequenced on an Illumina HiSeq4000 platform by the Majorbio Comp, following the manufacturer’s protocols. The raw data have been deposited in the NCBI Sequence Read Archive (accession number: SRP133889).

Raw reads from the sequencing machine were generated by base calling and saved in FASTQ format. Clean reads were generated by removing reads with adapters, reads where the number of unknown bases was more than 10%, and low-quality reads (the percentage of the low-quality bases with which value ≤5 was more than 50% in one read). Three biological replicates of each sample, showing correlation coefficient value of ≥0.95 were considered for subsequent gene expression analysis.

Twelve RNA libraries from the four plants were analyzed by using the RNA-Seq approach and comparative DEG profiling analysis (68). Approximately 43.64∼52.61 million raw RNA-Seq reads were produced from each individual sample. After filtering the dirty reads, 43.25∼52.10 million clean sequences were acquired and 95.2∼96.4% sequences were mapped to the reference genome of *Arabidopsis thaliana*. On the basis of these mapped reads, gene expression levels were calculated by using the reads per kb per million reads (RPKM) method (68).

### Real-time quantitative PCR

WT (Col-0), *flu*, flu,abi4 and *flu,ex1,ex2* mutants and *35S:ABI4-GFP/flu/abi4-2* complemented plants were grown for 21 days under continuous light, shifted to the dark for 8 h, and reexposed to light for 30 min. *ABI4*, *XCP1*, *AED3*, *F8H*, *LRX2*, *TMM*, *BCA1*, *FLU*, *PPCK1*, *SUS1*, *M17*, *NAS3*, and *CYP710A1* expression levels in above samples were detected by quantitative real-time PCR analysis. The cDNA was amplified by using SYBR Premix Ex Taq (TaKaRa). The *Ct* (threshold cycle), defined as the PCR cycle at which a statistically significant increase of reporter fluorescence was first detected, was used as a measure for the starting copy numbers of the target gene (57). Three technical replicates were performed for each experiment. *ACTIN1* gene (At2g37620) was used as internal controls. The expression levels of the WT seedlings in darkness are normalized to 100%. All primers are shown in Table S1.

### Antibody generation and Western blotting

Five epitopes of ABI4 protein were synthesized chemically. The polyclonal antibodies against these epitopes were generated by inoculation of mice and purified by Abmart Comp. The immune specificity of each antibody was verified by Western blotting. According to the Western blots (Fig. S4, *A* and *B*), the antibody against epitope#11 was selected for the following experiments.

Arabidopsis nuclear extracts were prepared by following the ChIP method (69). Briefly, nuclei were purified from total protein extracts with Percoll extraction buffer (95% V/V Percoll, 0.25 M sucrose, 10 mM Tris–HCl, pH 8.0 10 mM MgCl_2_, 5 mM β-mercaptoethanol, 5 mM NaF, 1× protease inhibitor cocktail) and washed twice with nuclei resuspension buffer (10% glycerol, 50 mM Tris–HCl, pH 8.0 5 mM MgCl_2_, 10 mM β-mercaptoethanol, 5 mM NaF, 1× protease inhibitor cocktail) by 10 min of centrifugation at 12,000*g*. Then nuclei were lysised in nuclei lysis buffer [50 mM Tris–HCl, pH 8.0 10 mM EDTA, 1% SDS, 5 mM NaF, 1× protease inhibitor cocktail].

SDS-PAGE and Western blotting analysis of the extracts was processed according to the method as described previously (16). For Western blots, 50 μg nuclear proteins were loaded for each sample.

### ChIP-quantitative PCR assay

The chromatin samples for ChIP experiments were obtained following the methods by Saleh *et al.* (69). The plants seedlings were first cross-linked by formaldehyde, and the purified cross-linked nuclei were then sonicated to shear the chromatin into suitably sized fragments. The antibody that specifically recognizes the recombinant ABI4 was used to immuno-precipitate DNA/protein complexes from the chromatin preparation. The DNA in the precipitated complexes was recovered and analyzed by qPCR methods. The chosen primer combinations (Table S1) could amplify fragments of 90 to 200 bp within the promoters of *ZP*, *WRKY33*, *WRKY46*, *DRP* and *ACC6* genes. To ensure the reliability of ChIP data, the input sample and no antibody (NoAB) control sample were analyzed with each primer set. Rabbit control IgG was purchased from Abcam Comp. The results were quantified with a calibration line made with DNA isolated from cross-linked and sonicated chromatin (69).

### Co-immunoprecipitation (Co-IP) assay

*35S:EX1-GFP* transgenic plants were grown for 5 days in the dark before transfer to 1/2 MS medium containing 100 μM RB and incubation in white light (100 μmol·m^−2^·s^−1^) for 0 or 30 min. For the possible interaction between ABI4 and EX1, nuclear proteins were extracted from the seedlings in Co-IP buffer (100 mM NaCl, 10 mM Tris–HCl, 10 mM MgCl_2_, 1 mM EDTA, 5% [v/v] glycerol, 1% [v/v] Triton X-100, 0.4% [v/v] NP-40 and 1 × complete protease inhibitor cocktail, pH 7.5). Then the proteins were immuno-precipitated with 20 μl anti-GFP–agarose beads or anti-ABI4–agarose beads by gentle shaking for 1 h at 4 °C in the dark (15). The pellets were washed with wash buffer four times before subjecting the immuno-precipitates to SDS-PAGE separation followed by immuno-blotting with mouse anti-GFP antibody (Abcam Comp.) or the anti-ABI4 antibody.

### Statistics analysis

All experiments were repeated three to five times, and average values were presented and the standard deviations (n ≥ 3) were shown. Student’s *t* test was used for comparison between different treatments. A difference was considered to be statistically significant when *p* <0.05. Statistically significant difference among plant strains were determined by one way ANOVA (Duncan’s multiple range test; *p* < 0.05).

## Data availability

Experimental raw data are available to be shared upon request to the corresponding authors.

## Supporting information

This article contains supporting information.

## Conflict of interest

The authors declare that they have no conflicts of interest with the contents of this article.

## Supporting information

Data sheet S1

Data sheet S2
