# Supplementary material for: Singlet oxygen induces cell wall thickening and stomatal density reducing by transcriptome reprogramming
Source: J Biol Chem. 2023 Nov 20;299(12):105481. doi: 10.1016/j.jbc.2023.105481 (PMC10731243; doi:10.1016/j.jbc.2023.105481)
Supplement: Supporting Figures S1–S13 and Table S1 [file mmc3.docx]

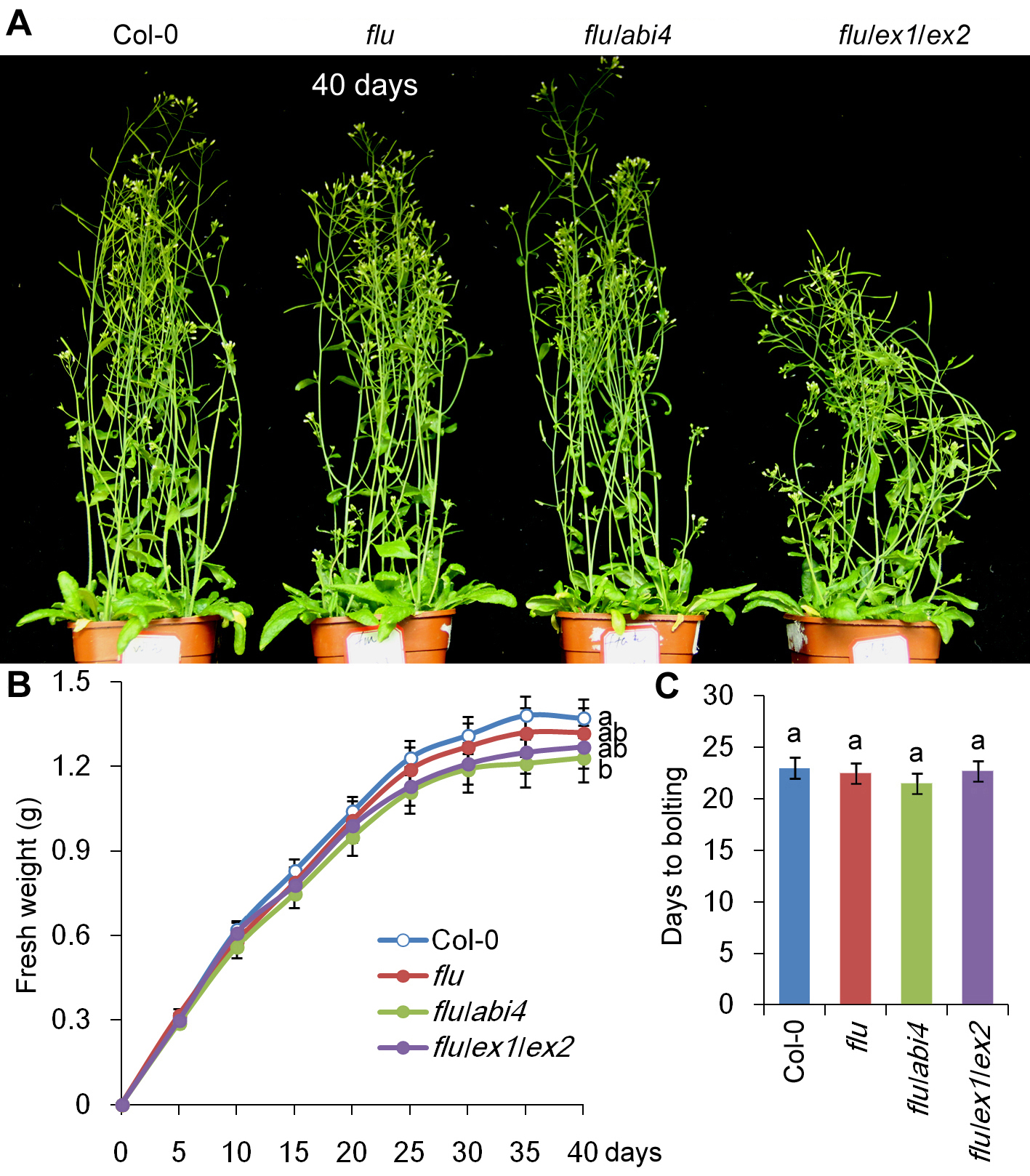


**Figure S1. All four lines of plants grow normally under continuous light.** *A*, 40-day-old wild-type (Col-0), *flu*, *flu*/*abi4* (*flu*/*abi4-104*) and *flu*/*ex1*/*ex2* seedlings grown under continuous light. *B*, 40-day-old seedlings grown under continuous light. *C*, Increasing in fresh weight during 40 days of growth under continuous light. *D*, Flowering times of all four lines of plants grown under continuous light. Error bars show standard deviations (*n* = 3). Different lowercase letters indicate significant differences at 0.05 (*P* < 0.05) levels.


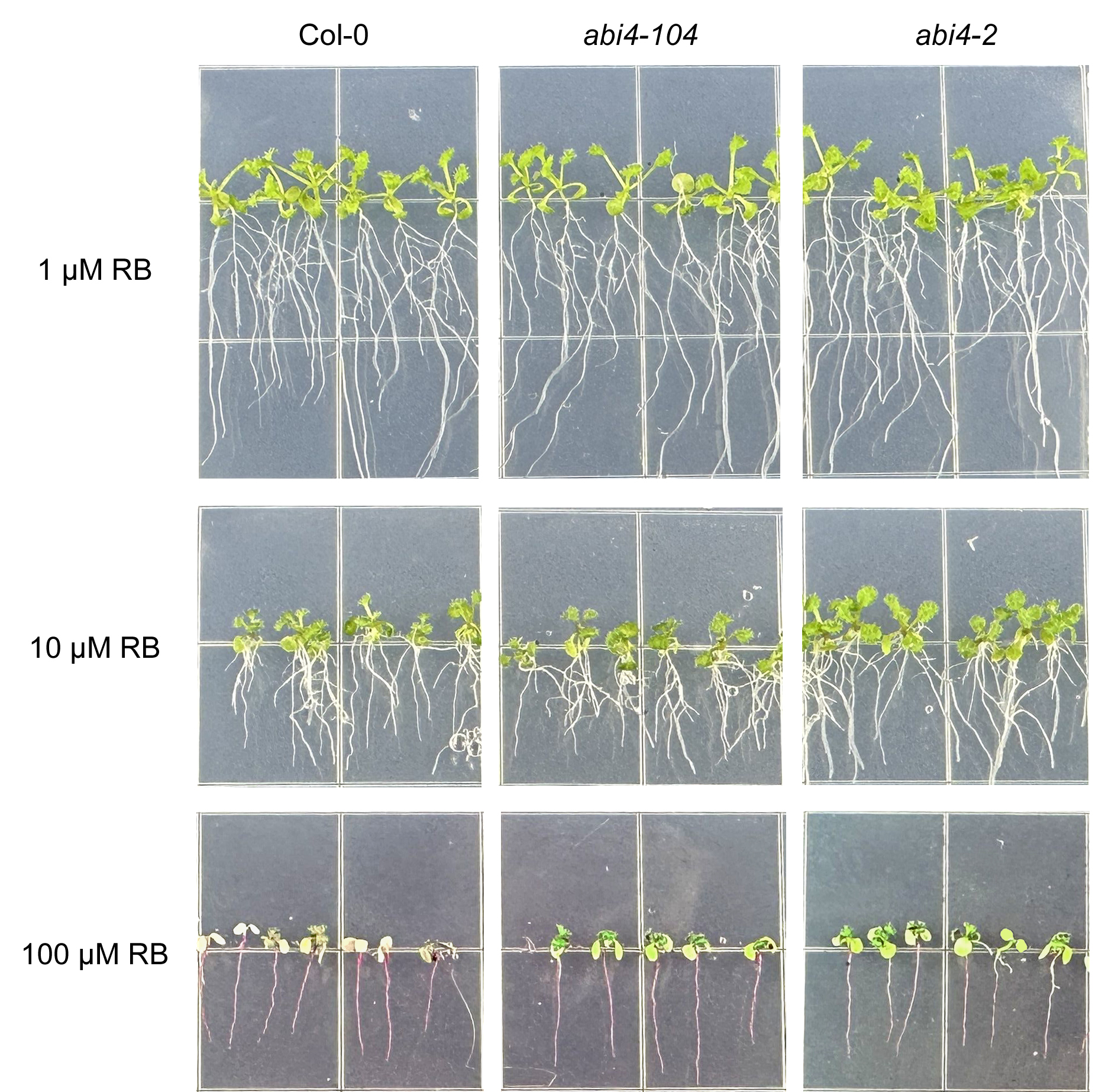


**Figure S2. The *abi4* mutants are hyposensitive to high-level RB treatments.** Wild-type (Col-0), *abi4-104* and *abi4-2* seedlings were grown on 1/2 MS medium under 16-h light (100 μmol ∙ m^-2^ ∙ s^-1^) / 8-h dark cycles without RB for 5 days and then transferred to 1/2 MS medium with 1, 10 or 100 μM RB for additional 6 days.


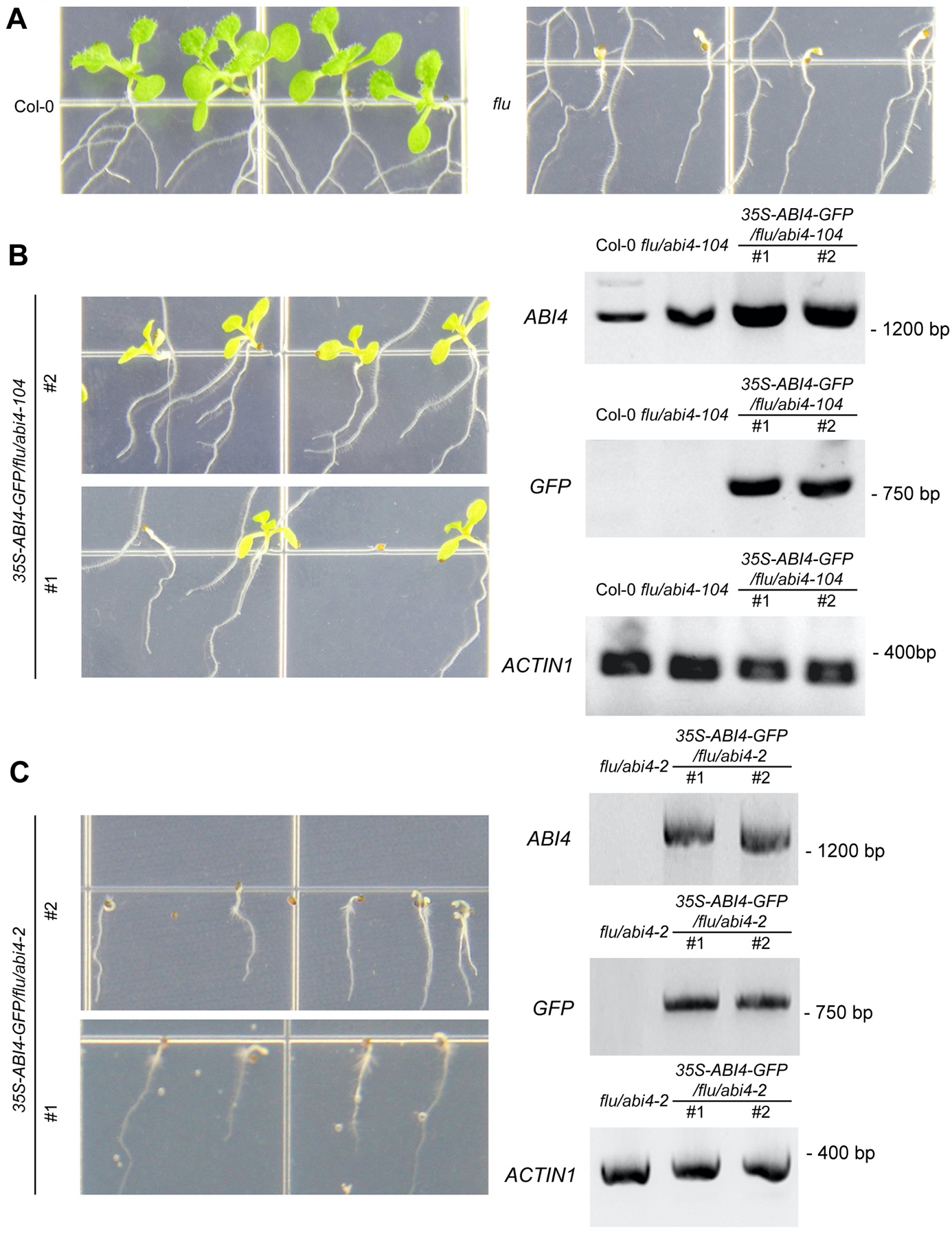


**Figure S3. *35S:ABI4*/*flu*/*abi4* complemented lines show growth arrest and photobleaching under light / dark cycles.** *A*, Phenotypes of wild-type (Col-0) and *flu* mutant. *B*, Phenotypes and *ABI4*, *GFP* and *ACTIN1* expression levels of *35S-ABI4-GFP/flu/abi4-104* (two complemented lines). *C*, Phenotypes and *ABI4*, *GFP* and *ACTIN1* expression levels of *35S:ABI4-GFP/flu/abi4-2* (two complemented lines). 11-day-old seedlings grown under light / dark cycles are shown. Transcript levels of *ABI4*, *GFP* and *ACTIN1* genes were detected by PCR.


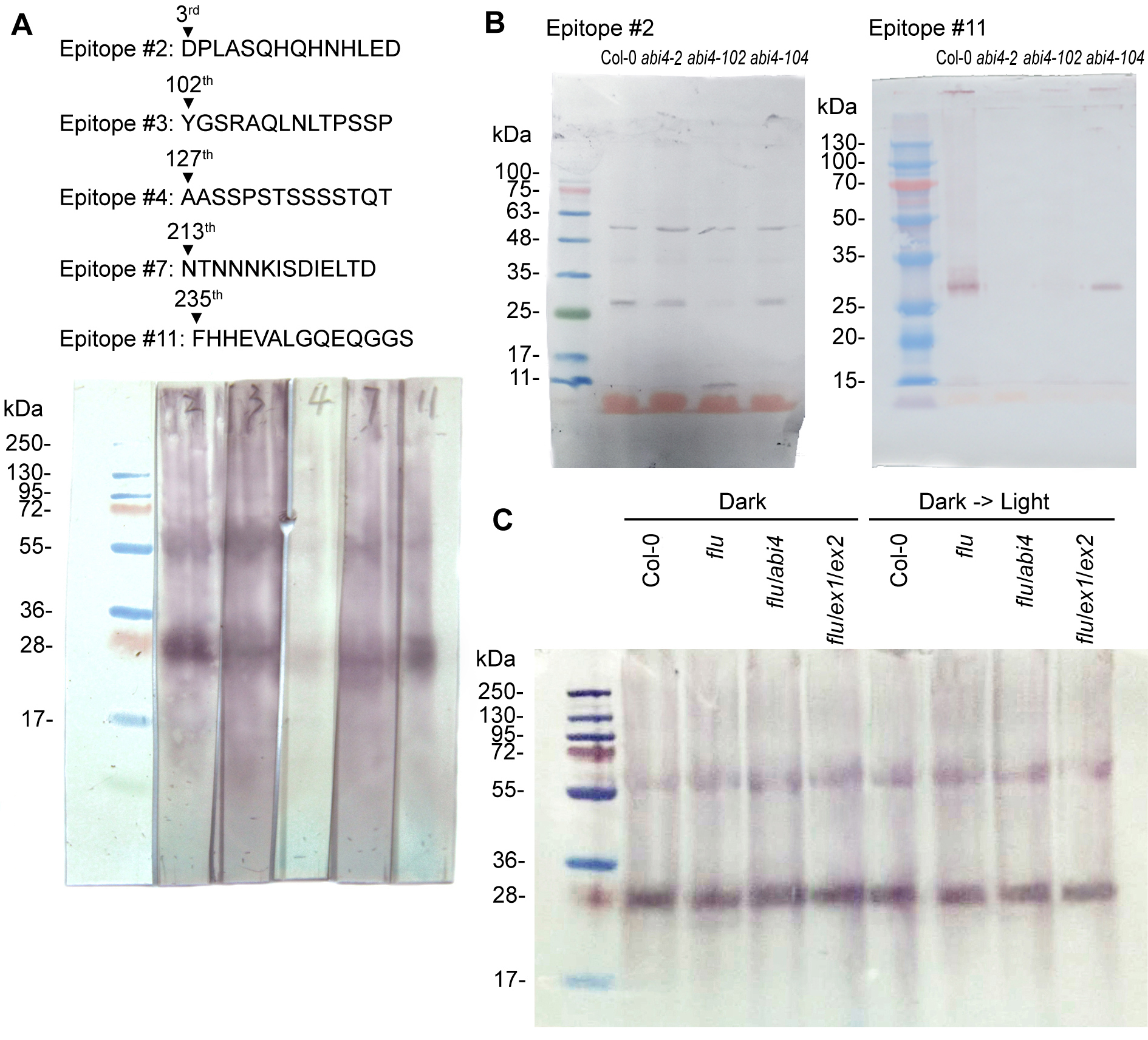


**Figure S4. Generation of antibodies against ABI4.** *A*, Immune specificity of the antibodies. Five epitopes of the ABI4 protein were synthesized chemically. The polyclonal antibodies against these epitopes generated by inoculation in mouse. The immune specificity of each antibody was verified by Western blotting of nuclear extracts from 21-day-old wild-type *Arabidopsis* seedlings. The predicted molecular weight of ABI4 was 35.68 kDa; however, only a 28-kDa band could be recognized by the antibodies, which might be correlated with its surface charge density or protein structure and thus requires further study. *B*, Western blotting of nuclear extracts from 21-day-old wild-type (Col-0), *abi4-102* (W to STOP at codon 80), *abi4-104* (single nucleotide substitution at codon 69 leading to missense E to K), and *abi4-2* (T-DNA insertion at codon 152) mutant seedlings with the antibody against epitope#2 or epitope#11. *C*, Western blotting of nuclear extracts from the wild-type (Col-0), *flu*, *flu*/*abi4* (*flu*/*abi4-104*), and *flu*/*ex1*/*ex2* plants grown for 21 days under continuous light, transferred to the dark for 8 h (Dark), and in some cases re-exposed to light for 30 min (Dark -> Light).


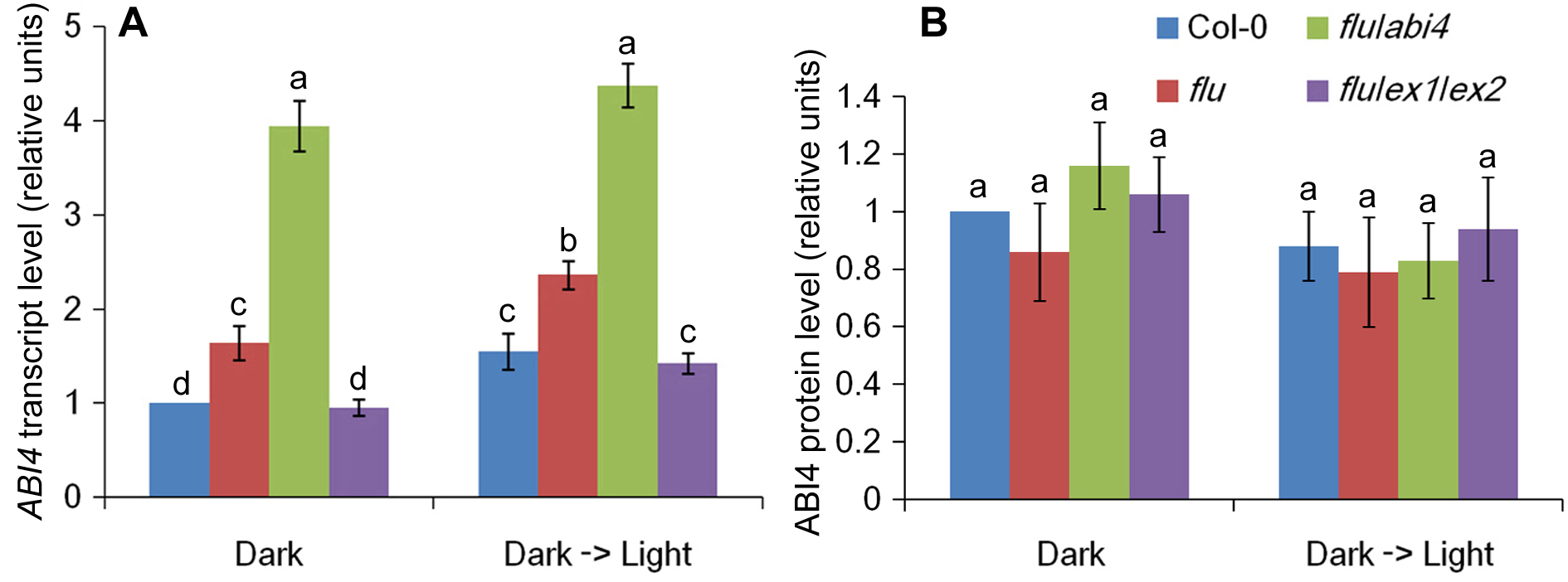


**Figure S5. Responses of *ABI4* gene and ABI4 protein to ^1^O_2_ signals.** Wild-type (Col-0), *flu*, *flu*/*abi4* (*flu*/*abi4-104*), and *flu*/*ex1*/*ex2* plants were grown for 21 days under continuous light, transferred to the dark for 8 h (Dark), and in some cases re-exposed to light for 30 min (Dark -> Light). Gene expression levels were detected by quantitative real-time PCR. Protein levels were detected by Western blotting (as shown in Fig. S4*C*). The levels of the wild-type seedlings in 8-h dark were normalized to 100%. Error bars show standard deviations (*n* = 3). Different lowercase letters indicate significant differences at the 0.05 (*P* < 0.05) level. *ABI4* transcript was upregulated in *flu*/*abi4* seedlings, possibly in an attempt to compensate for the non-functional ABI4.


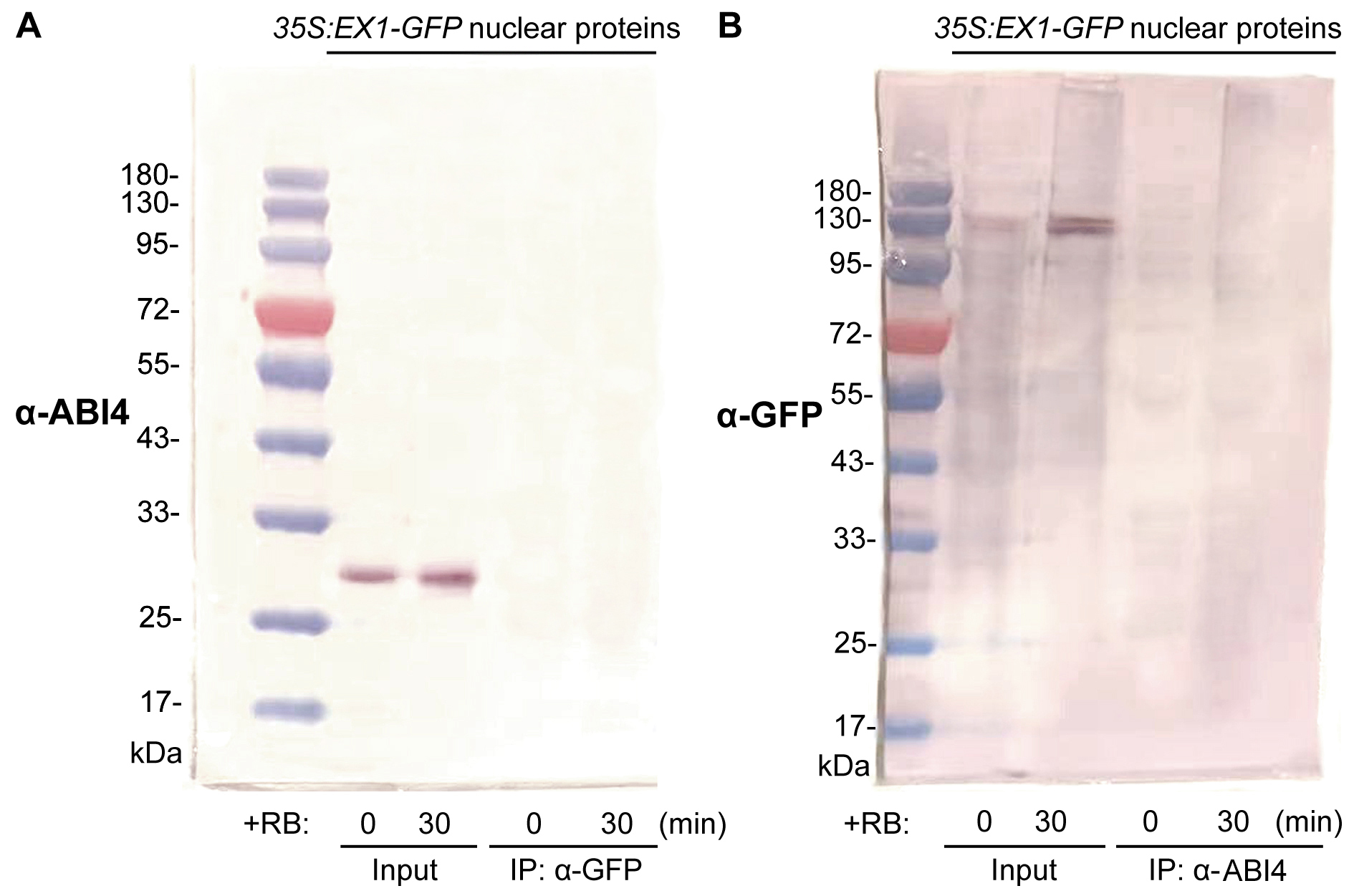


**Figure S6. EX1 does not bind to ABI4 directly after RB treatment.** *35S:EX1-GFP* transgenic plants were grown for 5 days in the dark before transfer to 1/2 MS medium containing 100 μM RB and incubation in white light (100 μmol ∙ m^-2^ ∙ s^-1^) for 0 or 30 min. Nuclear proteins were extracted from the seedlings in Co-IP buffer. Then the proteins were immuno-precipitated with 20 μL anti-GFP–agarose beads (IP: α-GFP) or anti-ABI4–agarose beads (IP: α-ABI4). The pellets were subjected to SDS-PAGE separation followed by immuno-blotting with anti-GFP antibody or anti-ABI4 antibody. EX1-GFP was present in the nucleus upon 30-min RB and light treatment. EX1 could not immuno-precipitated with ABI4 (*A*); ABI4 could not immuno-precipitated with EX1 (*B*).


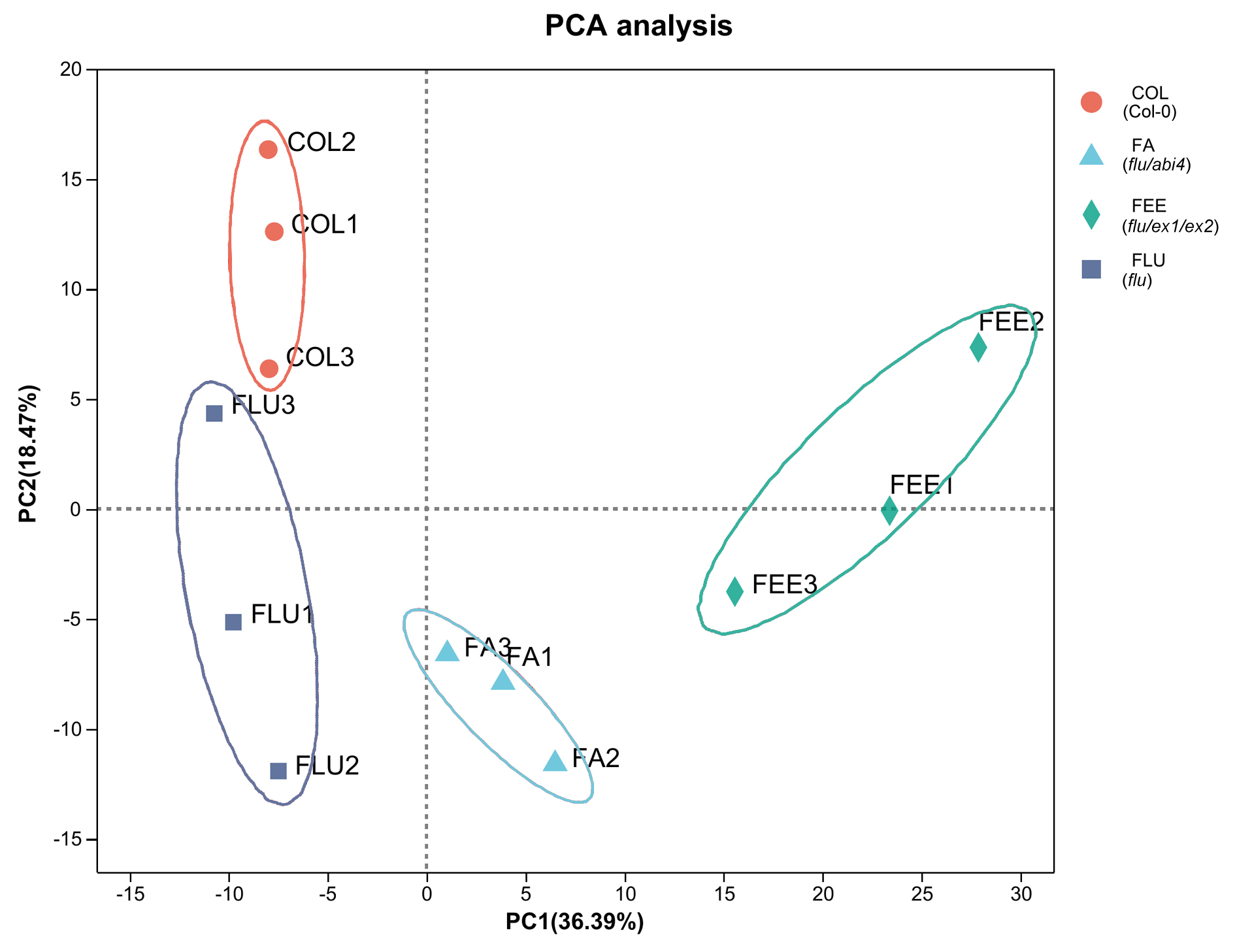


**Figure S7. Principal component analysis (PCA) of differentially expressed genes.** Wild-type (Col-0), *flu*, *flu*/*abi4* (*flu*/*abi4-104*) and *flu*/*ex1*/*ex2* plants were grown for 21 days under continuous light, transferred to the dark for 8 h, and then re-exposed to light for 30 min. Equal quantities of RNA from three independent biological replicates of each plant were pooled for RNA-seq.


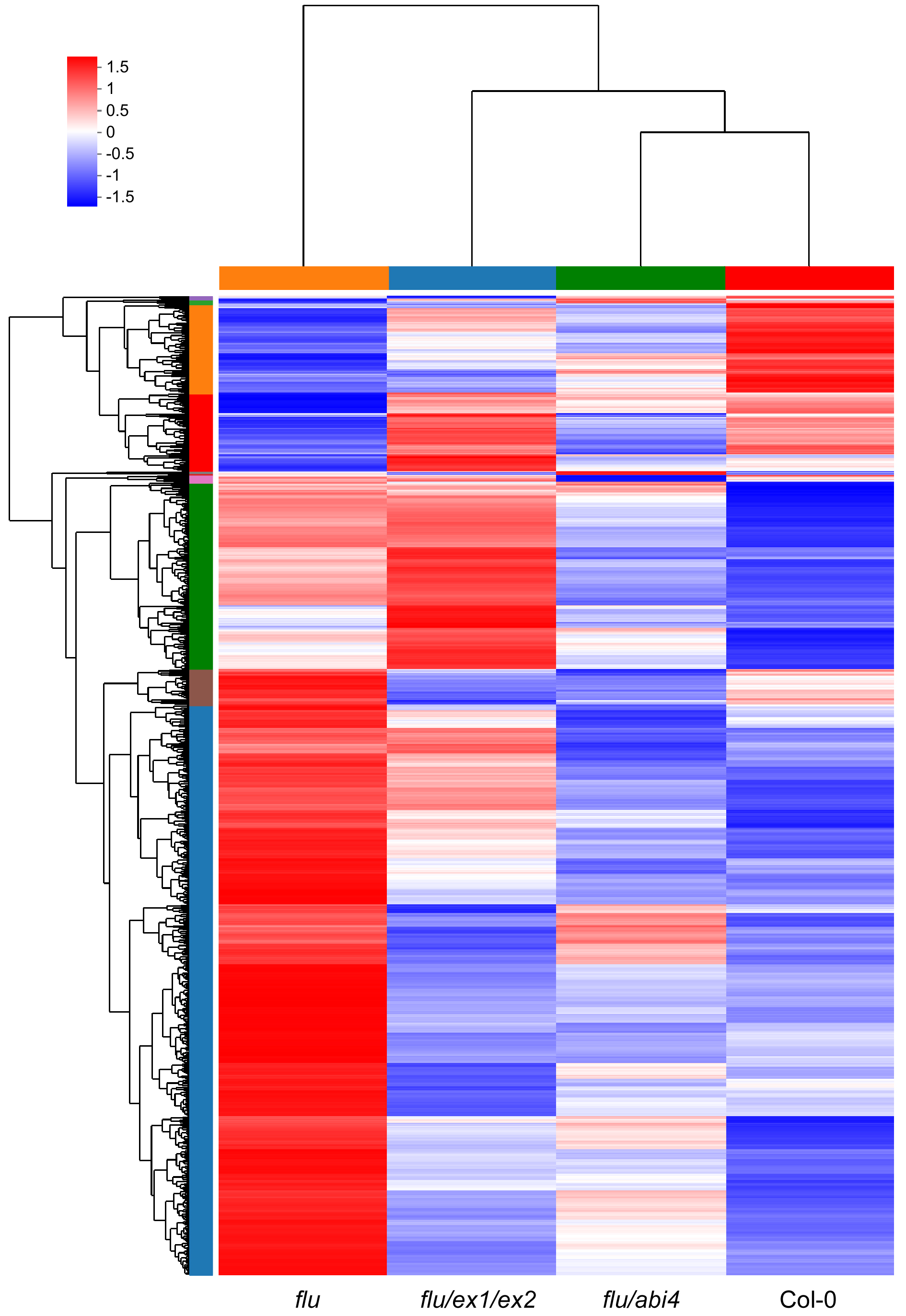


**Figure S8. Heatmaps of differentially expressed genes.** Wild-type (Col-0), *flu*, *flu*/*abi4* (*flu*/*abi4-104*) and *flu*/*ex1*/*ex2* plants were grown for 21 days under continuous light, transferred to the dark for 8 h, and then re-exposed to light for 30 min. Equal quantities of RNA from three independent biological replicates of each plant were pooled for RNA-seq. The log_10_ ^FPKM value^ was colored by Cluster 3.0 (red for up-regulated, blue for down-regulated). FPKM, Fragments per kilobase of exon model per million mapped fragments.


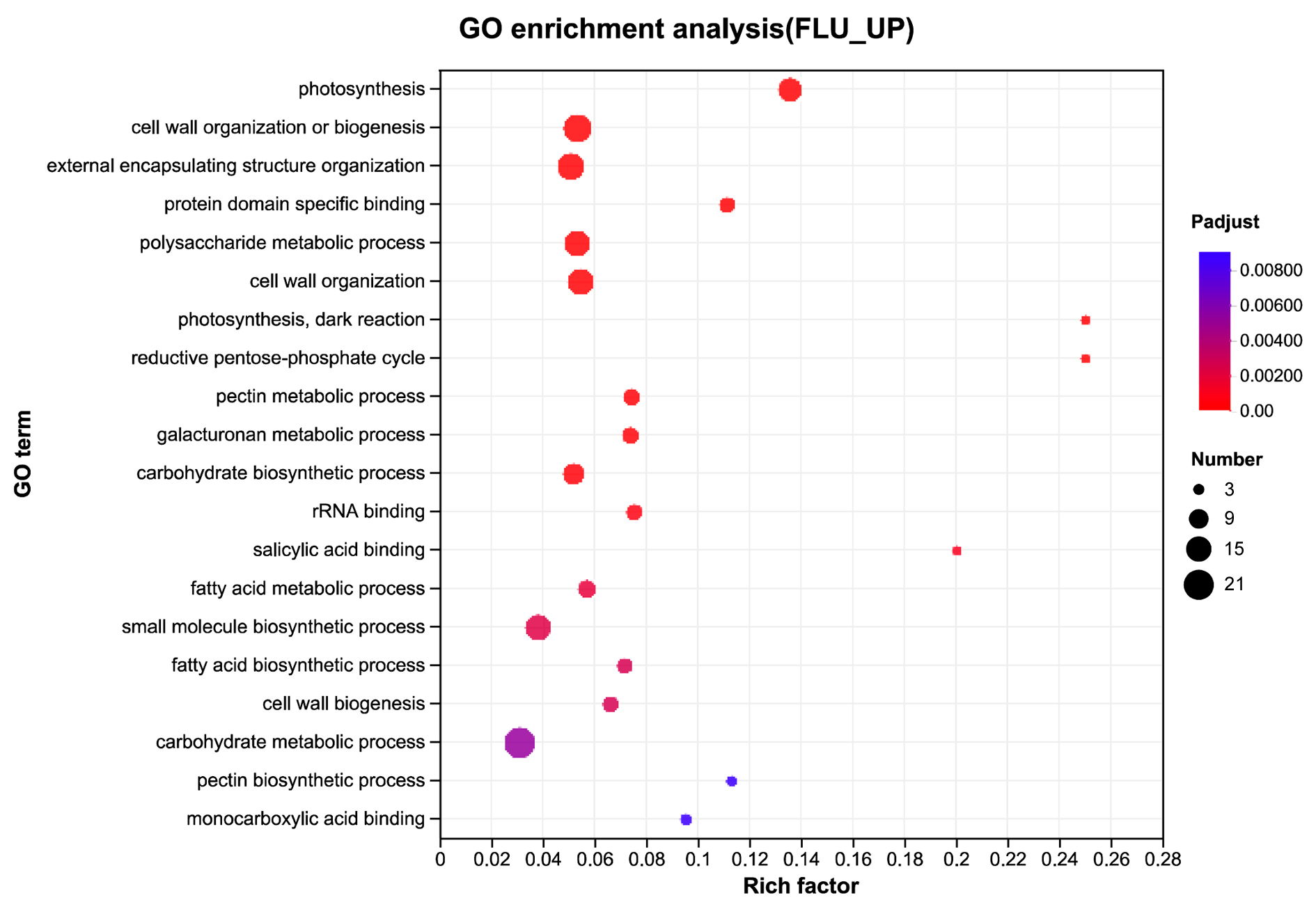


**Figure S9. Gene Ontology (GO) enrichment analysis of ^1^O_2_-induced genes.** Wild-type (Col-0), *flu*, *flu*/*abi4* (*flu*/*abi4-104*) and *flu*/*ex1*/*ex2* plants were grown for 21 days under continuous light, transferred to the dark for 8 h, and then re-exposed to light for 30 min. Equal quantities of RNA from three independent biological replicates of each plant were pooled for RNA-seq. Genes with a 2-fold or greater transcript level than the control were considered to be significantly upregulated. Among them, 200 transcripts were ^1^O_2_-induced genes in *flu* specifically and subjected to GO enrichment analysis.


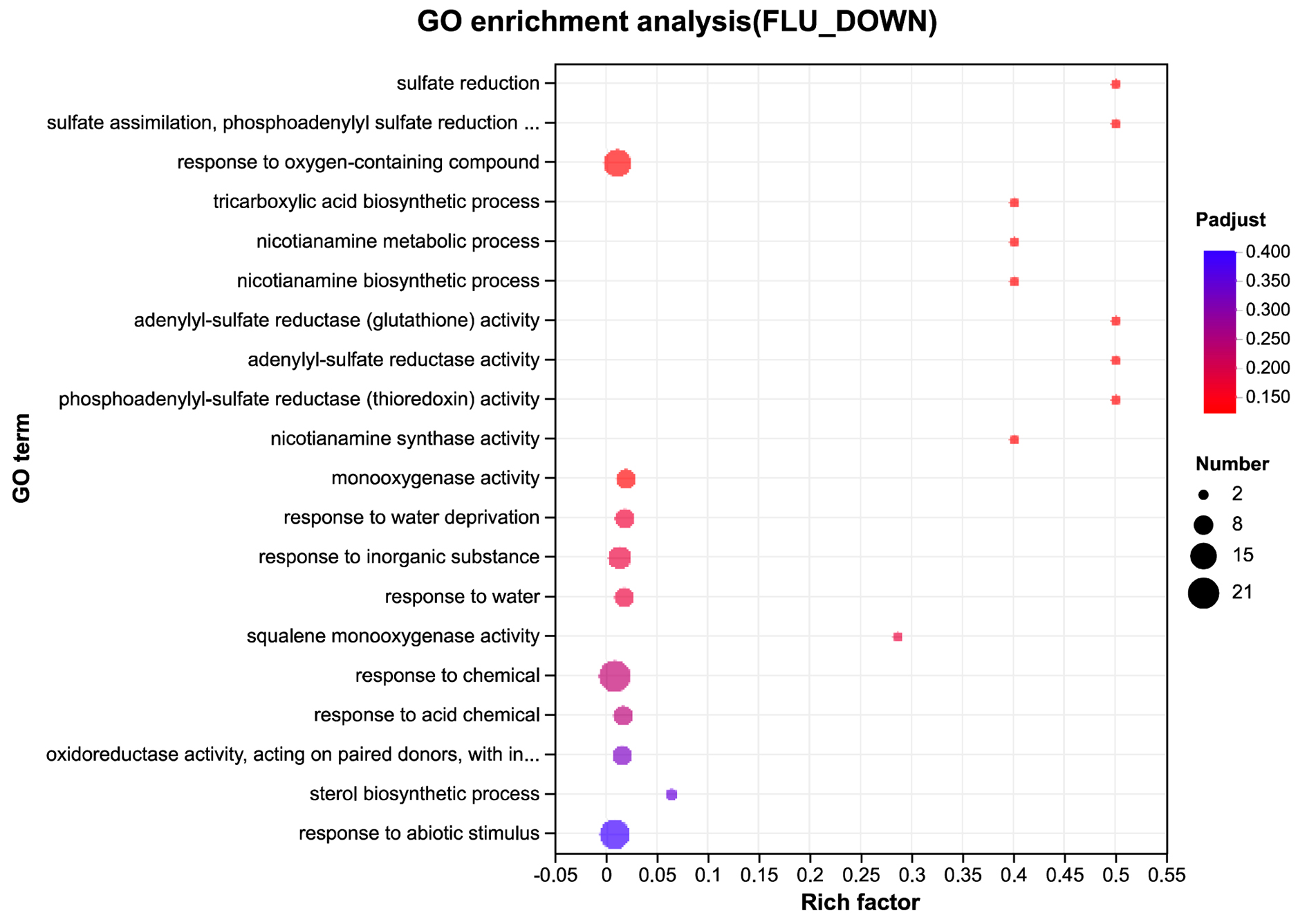


**Figure S10. Gene Ontology (GO) enrichment analysis of ^1^O_2_-repressed genes.** Wild-type (Col-0), *flu*, *flu*/*abi4* (*flu*/*abi4-104*) and *flu*/*ex1*/*ex2* plants were grown for 21 days under continuous light, transferred to the dark for 8 h, and then re-exposed to light for 30 min. Equal quantities of RNA from three independent biological replicates of each plant were pooled for RNA-seq. Genes with a 2-fold or lower transcript level than the control were considered to be significantly downregulated. Among them, 110 transcripts were ^1^O_2_-repressed genes in *flu* specifically and subjected to GO enrichment analysis.


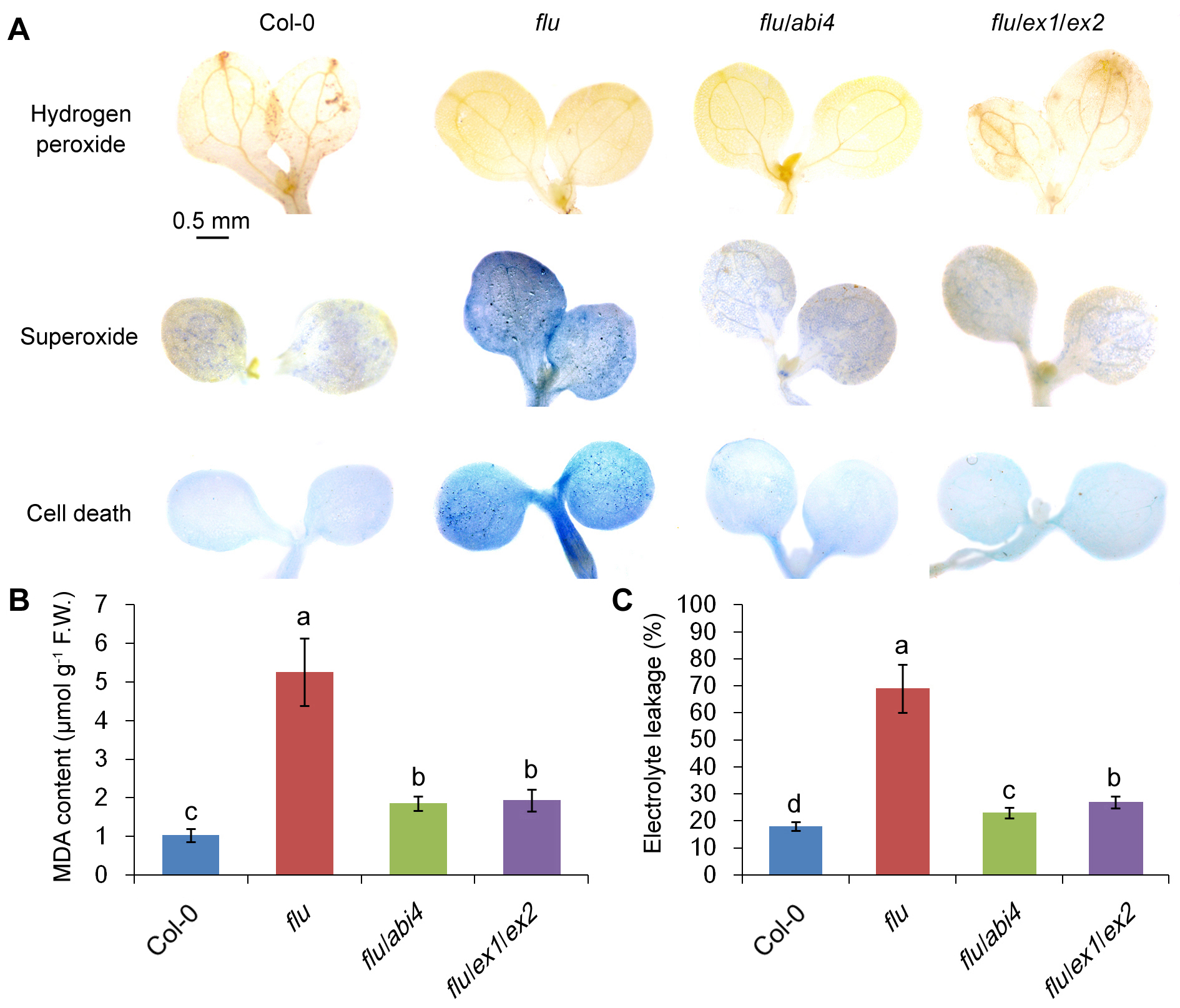


**Figure S11. Light / dark cycles induce ROS accumulation, lipid peroxidation and cell death in *flu* mutant.** H_2_O_2_, O_2_∙^-^ and cell death staining (*A*), malondialdehyde (MDA) content (*B*) and electrolyte leakage (*C*) of 7-day-old wild-type (Col-0), *flu*, *flu*/*abi4* (*flu*/*abi4-104*) and *flu*/*ex1*/*ex2* seedlings grown under light / dark cycles. H_2_O_2_ and O_2_∙^-^ levels were visually detected with 3,3-diaminobenzidine (DAB) and nitro blue tetrazolium (NBT) respectively. The photobleaching (cell death) was visually detected by trypan-blue staining. F.W., fresh weight. Error bars show standard deviations (*n* = 3). Different lowercase letters indicate significant differences at 0.05 (*P* < 0.05) levels.


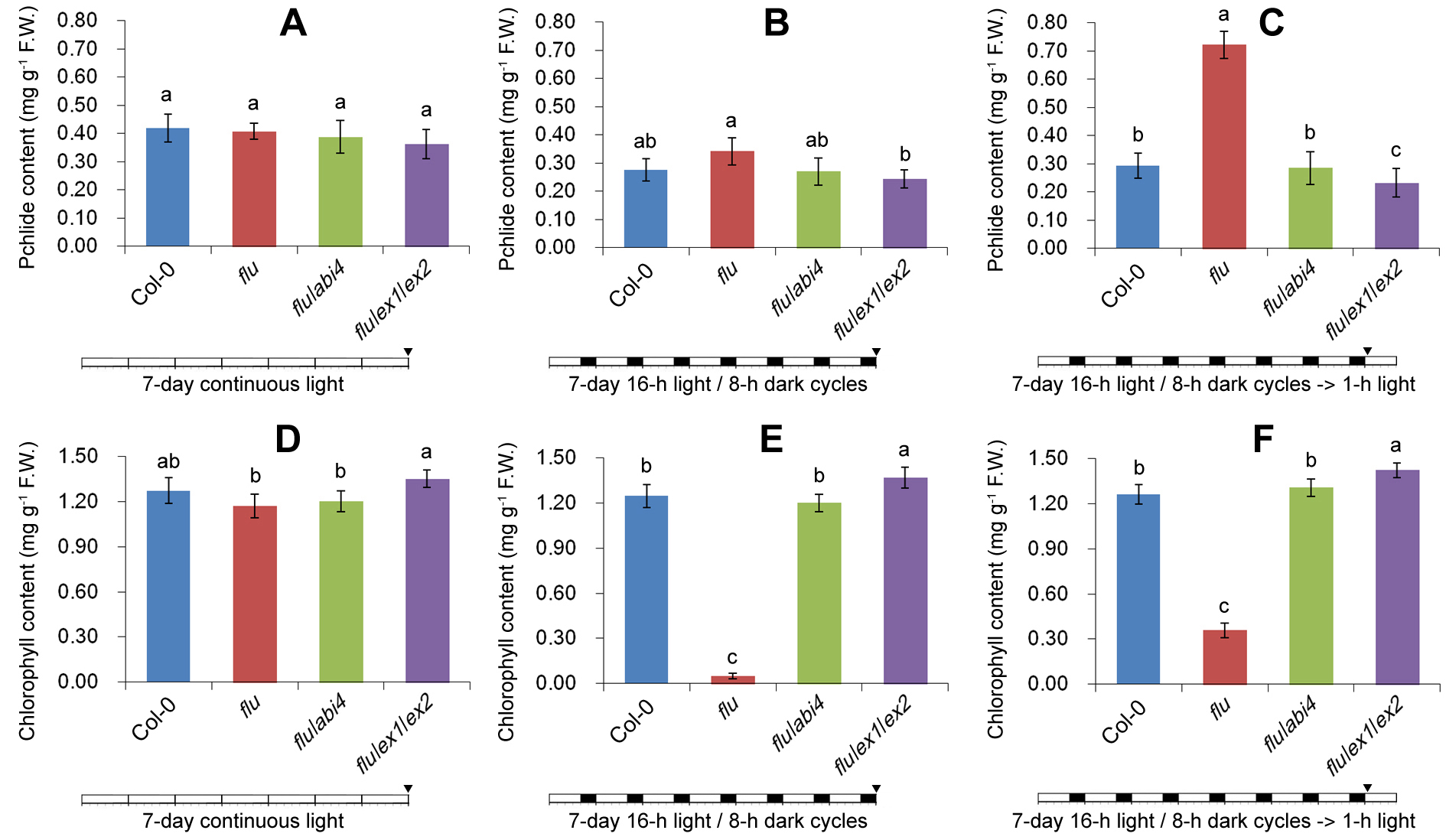


**Figure S12. Photobleaching and Pchlide accumulation in *flu* mutant grown under light / dark cycles.** Wild-type (Col-0), *flu*, *flu*/*abi4* (*flu*/*abi4-104*) and *flu*/*ex1*/*ex2* plants were grown for 7 days under continuous light (*A*, *D*) or light / dark cycles (*B*, *E*), and in some cases re-exposed to light for 1 h (*C*, *F*). Then their Pchlide contents (*A–C*) and chlorophyll contents (*D–F*) were determined. F.W., fresh weight. Error bars show standard deviations (*n* = 3). Different lowercase letters indicate significant differences at 0.05 (*P* < 0.05) levels.


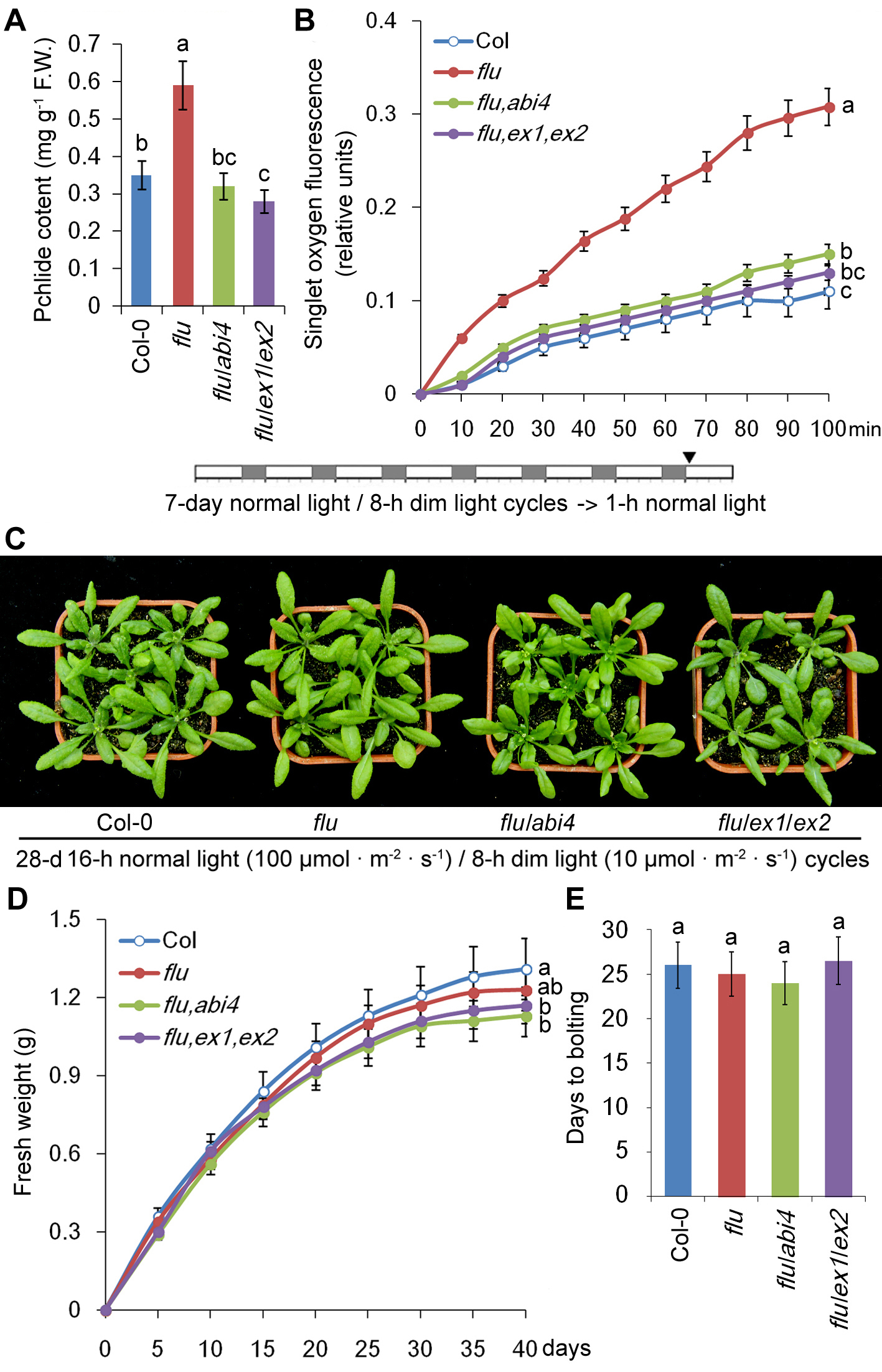


**Figure S13. The *flu* mutant grows normally under normal light / dim light cycles.** Wild-type (Col-0), *flu*, *flu*/*abi4* (*flu*/*abi4-104*) and *flu*/*ex1*/*ex2* plants were grown for 7 days under 16-h normal light (100 μmol ∙ m^-2^ ∙ s^-1^) / 8-h dim light (10 μmol ∙ m^-2^ ∙ s^-1^) cycles and then re-exposed to normal light for 1 h. Then their Pchlide contents (*A*) and singlet oxygen generation ratios (*B*) were determined. F.W., fresh weight. *C*, 28-day-old seedlings grown under 16-h normal light / 8-h dim light cycles. *D*, Increasing fresh weight during 40 days of growth under 16-h normal light / 8-h dim light cycles. *E*, Flowering times of all four lines of plants grown under 16-h normal light / 8-h dim light cycles. Error bars show standard deviations (*n* = 3). Different lowercase letters indicate significant differences at the 0.05 (*P* < 0.05) level.

**Table S1. List of primers for characterizing Arabidopsis genes.**

| Gene | Location | L primer | R primer |
| --- | --- | --- | --- |
| *ZP* | At5g04340 | CACGCAACTTCCTTCCTCTTCA | GACACGCTACTACTCACACCGC |
| *ZP-ChIP-1* | At5g04340 | GTTGTGCTTTAAATTCTGTCTTCTCTT | TTAACCATTTTATTAAGTTTATGGTTG |
| *ZP-ChIP-2* | At5g04340 | AGGTTACGTTGATATTGATGGGTTT | AGCTCTCACTTAGGTGTTTTGCTGA |
| *WRKY33* | At2g38470 | GAAGCAAAGAGATGGAAAGGGG | AGGCGTGTGAAAAAACAAATGA |
| *WRKY33-ChIP-1* | At2g38470 | GACTAGTTAAGGAAACAACAAACTG | AAATGTTCTTATTGAGATGTCAGGA |
| *WRKY33-ChIP-2* | At2g38470 | TGAAGATGATGAAAGCATTGAGCCG | TGATTAGAAGAAGAAGGAGAAGAGA |
| *WRKY46* | At2g46400 | CGAAACTCACATCTCCCACCAA | GCTACGACCACAACCAATCCTG |
| *WRKY46-ChIP-1* | At2g46400 | TGTGAATGAAAAAGACACATTGGTATA | AGATCGAAATTAATCAAATTTTAGTGG |
| *WRKY46-ChIP-2* | At2g46400 | TTTTATCAGAAACGAGTAACCCCTA | CAATCTTCGACCAGAAGTTGTGTGG |
| *DRP* | At1g66090 | TGTGCTCGTAAAACAGAGGAGGA | CCAAAACATGTAGATGCAATGGA |
| *DRP-ChIP-1* | At1g66090 | CCTAAAATCATGTACAATAGAGGTGTT | TATCCAATCAGAAATGAATATGTGTCA |
| *DRP-ChIP-2* | At1g66090 | GTTTTGTATTTATTAAACAAACTATTGG | CACTTTTTTTTATTTTTTTTCATACTCC |
| *ACC6* | At1g11280 | AGATGTAGGAAATGGAACAGGGC | CCAAAATCACAAAAATGGAAAGG |
| *ACC6-ChIP-1* | At1g11280 | TATGGTGGAGATAAAGAACGAGGTG | CAGTTGTATTCAGCAATCCAAAAGA |
| *ACC6-ChIP-2* | At1g11280 | GCTCTGTTTTTCCTTTCTTTCCCA | AACTCCATCTTACATCAACCCTGT |
| *ABI4* | At2g40220 | TTTAGCTTCCCAACATCAACACAA | ATTCCCATACAACTTTCCACCATC |
| *ABI4-ORF* | At2g40220 | TTCTTGATTCGTCCTCTCCACTAT | TACAACTTTCCACCATCTCCTCC |
| *XCP1* | At4g35350 | GCTATGGCTTTTTCTGCACCA | GTCAAATCCGCAAACTCGTTC |
| *AED3* | At1g09750 | GACTCCCATCGACTCACCTACC | ACGACACATTCACCTGCTTCCT |
| *F8H* | At5g22940 | CGTCTCTCCCATCTCGTGCT | TTGACTTCCATTTTTCCCCG |
| *LRX2* | At1g62440 | GATGTGTGGCTCTTTTGGGAG | GGACTTTGTGTTGTGGGTGGA |
| *TMM* | At1g80080 | TTCGAACCTAAACCGCATTCA | TCCCAAAAAACCATTCTCCCT |
| *BCA1* | At3g01500 | CTATCGCTTTCGTTATCCTCGTTC | GGGTTGGTTTCGTATTTCTCCTTC |
| *FLU* | At3g14110 | TGCTCTTATTGGAGCCACCGT | GCCTCTTGCAGCCTTCTTTTC |
| *PPCK1* | At1g08650 | GAACAAGCATTAAGGCACCCAT | TCAGAAGCAGCCACAACAAACA |
| *SUS1* | At5g20830 | GAGACAGGAGGAAAGAGTCAAAGG | GAAATCAGCAAGAGTATCAGCAGC |
| *M17* | At2g41260 | TCTCGTTCTCTTGGCTTTGCTC | GGTTTACCGTCATGTTTTTGCG |
| *NAS3* | At1g09240 | CCAACCCCACGCCAATAAATA | TGACCTTCGGCTAGACCACAA |
| *CYP710A1* | At2g34500 | GGCAGGCAAGACTACAAAGCA | ATTGGAGGGACGAAGAAAGGG |
| *ACTIN1* | At2g37620 | TGTTATTGTTTTGGGGGACTGG | TCTGCTGGAAGGTACTGAGGGA |
| *GFP* |  | CAGTGGAGAGGGTGAAGGTGAT | GGCAGATTGTGTGGACAGGTAA |
